# Covalently linked phosphate monoesters on alpha-polyglucans reduce substrate affinity of branching enzymes

**DOI:** 10.1016/j.carbpol.2025.123561

**Published:** 2025-04-04

**Authors:** Victoria Butler, Hanan Shaaban, Lilya Nasanovsky, Jessica K.V. White, Owen Hebb, Lynne Jones, Megan Whiting, Neije Mukherjee-Roy, Alina P. Montalbano, Carmen Elena Machado Vides, Felix Nitschke, Ian J. Tetlow

**Affiliations:** a Department of Molecular and Cellular Biology, College of Biological Science, University of Guelph, Ontario N1G2W1, Canada; b Botany Department, Faculty of Science, Ain Shams University, Cairo 11566, Egypt; c Department of Biochemistry, University of Texas Southwestern Medical Center, Dallas, TX 75390, USA

**Keywords:** Amylopectin, Glycogen, Phosphate, Branching enzymes, Starch, Glycogen phosphorylation, Starch phosphorylation

## Abstract

Starch and glycogen are α-polyglucans which represent important sources of long- and short-term cellular carbohydrate storage synthesized in living cells. Both polyglucans contain variable, but significant, levels of covalently bound phosphate monoesters whose biological role is likely connected to the regulation of turnover of these storage polymers by promoting water solubility. The amount of α-glucan-bound phosphate found in plant starch appears to be closely related to the average chain length of α-1,4-glucans, and inversely related to the frequency of α-1,6-branch linkages. The enzymes responsible for adding branches to linear α-1,4-glucan chains in starch and glycogen are 1,4-α-glucan: 1,4-α-glucan 6-glucosyl transferases (branching enzymes). In this study, glucan bound phosphate was shown to reduce the affinity of branching enzymes for α-glucan substrates. Plant starch branching enzymes and glycogen branching enzymes from various prokaryotic and eukaryotic sources showed reduced substrate affinities in native gels as the α-glucan phosphate content was increased. The substrate affinities of all branching enzymes tested showed an inverse linear relationship with α-glucan phosphate content. The possible biological significance of this phenomenon is discussed in relation to known models of starch structure in plants and specific glycogen storage diseases in mammals.

## 1. Introduction

Efficient carbohydrate storage and turnover are crucial elements of cellular carbon metabolism and underpin organism fitness. Glycogen and starch are the major forms of osmotically inert carbohydrate storage found in nature. In heterotrophic prokaryotic and eukaryotic organisms glycogen is a major form of short-term carbohydrate storage made available for immediate use in order to meet energy demands (Prats, Graham, & Shearer, 2018; Roach, Depaoli-Roach, Hurley, & Tagliabracci, 2012; Wilson et al., 2010). In autotrophic higher plants and green algae starch plays a central role in carbon storage in both the short-term over a diurnal cycle, and long-term for seed and tuber storage in plants, linking photosynthetic carbon fixation to nocturnal growth and development, and providing carbon stores for the next generation (Graf, Schlereth, Stitt, & Smith, 2010; MacNeill et al., 2017; Sulpice, Pyl, Ishihara, & Stitt, 2009). Despite both polyglucans being chemically identical, *i.e.*, composed of linear α-1,4-linked glucan chains and α-1,6-branch linkages, they possess structurally and physicochemically distinct properties that make them suited to their respective biological roles.

Glycogen is highly branched (~9–18 % of glucose (Glc) units are α-1,6-linked) and found as roughly spherical water-soluble particles containing as many as 55,000 Glc units, possibly arranged with up to twelve layers of α-1,6-branch linkages (Marchand et al., 2002), although studies with mouse liver glycogen suggest branching is random (Besford, Zeng, Ye, & Gray-Weale, 2016; Neoh et al., 2024). In eukaryotes, the smallest glycogen particles, termed β-granules, are approximately 20 nm in diameter, although larger assemblies (termed α-particles) are found in different organs (Drochmans, 1962; Liu & Gilbert, 2024; Prats et al., 2018). Eukaryotic glycogen biosynthesis occurs through the actions of a small group of enzymes in the cytosol: a dimeric proteinaceous primer called glycogenin (EC 2.4.1.186), glycogen synthase (GS, EC 2.4.1.11) which elongates α-glucan chains by transferring glucosyl units from UDP-glucose to the non-reducing end of an extant α-1,4-linked chain, and glycogen branching enzyme (GBE, EC 2.4.1.18) which introduces α-1,6-branch linkages with an average chain length (degree of polymerization, DP) of ~13 glucosyl units (Nitschke et al., 2013; Roach et al., 2012). The relatively simple pathway of glycogen biosynthesis belies a complex network of cellular regulation controlling glycogen turnover, such that the pathway is highly responsive to cellular Glc6P and ATP levels (Adeva-Andany, González-Lucán, Cristóbal Donapetry-García, Fernández-Fernández, & Ameneiros-Rodríguez, 2016; Jensen & Richter, 2012; Wilson et al., 2010). A key feature of the flexibility of glycogen mobilization in response to metabolic energy demands is its water-solubility and accessibility of α-glucan chains to the degradative enzymes glycogen phosphorylase (GP, EC 2.4.1.1) and glycogen debranching enzyme (GDBE, EC 2.4.1.25/3.2.1.33) to allow release of Glc units (in the form of Glc1P) into the cell (Gordon, Brown, & Brown, 1972; Newgard, Hwang, & Fletterick, 1989). Starch, on the other hand, is synthesized as water-insoluble granules which are orders of magnitude larger (0.1 to 100 μm) than glycogen particles (Jane, Kasemsuwan, Leas, Zobel, & Robyt, 1994; Jenkins, Cameron, & Donald, 1993). Branch points in amylopectin, the major component of starch, are clustered and represent approximately 5 % of total glucosyl residues (Bertoft & Tetlow, 2020; Inouchi, Glover, & Fuwa, 1987; Manners & Matheson, 1981; Thompson, 2000). The clustered α-1,6-linked branch points in amylopectin allow self-assembly of short and intermediate length α-glucan chains (DP 10–12) to form left-handed double helices which exclude free water (Cooke & Gidley, 1992; Gidley & Bulpin, 1987; Imberty, Buléon, Tran, & Pérez, 1991; Sarko & Wu, 1978) giving rise to higher order structures termed allomorphs which appear to be related to the biological functionality of the starch within a given plant organ, and are distinguished using wide-angle *X*-ray diffraction (Damager, Engelsen, Blennow, Møller, & Motawia, 2010; Pfannemüller, 1987; Salman et al., 2009). Long term storage starches *e.g.*, found in cereal endosperms are made up of closely packed double helices and show the A-allomorph. Starches which are prone to frequent turnover *via* amylolysis, *e.g.* transient leaf starch and tuber and root starches, show a more open, hydrated structure and are termed B-allomorph (Buléon, Colonna, Planchot, & Ball, 1998; Tetlow & Butler, 2023). The initiation and subsequent synthesis of this architecturally complex polymer takes place inside specialized organelles termed plastids through the action of many enzymes and regulatory proteins (Goren, Ashlock, & Tetlow, 2018; Butler & Tetlow, 2024; Pfister & Zeeman, 2016). The major classes of enzymes involved in starch biosynthesis are broadly similar to those of glycogen synthesis but generally consist of multiple isoforms of each class, and include starch synthases (EC 2.4.1.21), starch branching enzymes (SBE, EC 2.4.1.18) and debranching enzymes (isoamylases, EC 3.2.1.68), these latter enzymes playing a critical biosynthetic role in determining the semi-crystalline, water-insoluble properties of amylopectin (Ball et al., 1996; James, Robertson, & Myers, 1995; Sim et al., 2014).

A common feature of both starch and glycogen is the presence of varying amounts of phosphate monoesters covalently attached to glucosyl residues (Blennow & Engelsen, 2010; Donohue, Kuchtova, Vander Kooi, & Gentry, 2021; Nitschke & Schmieder, 2021). In plant starches these phosphate groups are found in the branched amylopectin component of the granule, and not in amylose, which is synthesized within an extant amylopectin matrix (Blennow, Engelsen, Nielsen, Baunsgaard, & Mikkelsen, 2002; Kozlov, Blennow, Krivandin, & Yuryev, 2007; Samec, 1914). Two dikinases are responsible for the covalent monoesterification of the β-phosphate from ATP at C6 and C3 positions of the pyranosidic glucose residues of amylopectin. α-Glucan water dikinase (GWD, EC 2.7.9.4) incorporates C6 phosphate groups on longer α-glucan chains of 28–30 DP (Mikkelsen, Baunsgaard, & Blennow, 2004; Ritte et al., 2002; Ritte et al., 2006) and phospho-glucan water dikinase (PWD, EC 2.7.9.5) adds phosphate at the C3 position of pre-phosphorylated α-glucans which represents approximately 20–30 % of total starch phosphate (Baunsgaard et al., 2005; Kötting et al., 2005; Ritte et al., 2006). Despite early reports of glucosyl 2-phospho monoesters (C2) in potato starch (Tabata & Hizukuri, 1971), further evidence for C2 phosphate monoesterification in plants is not very strong (Donohue et al., 2021). The mechanism underlying glycogen phosphorylation is currently unknown. Glycogen from different tissues and organs contains variable levels of phosphate (Fontana, 1980; Kirkman & Whelan, 1990; Lomako, Lomako, Kirkman, & Whelan, 1994) and is esterified at C6, C3, and C2 positions by an unknown enzyme or process (DePaoli-Roach et al., 2015; Nitschke et al., 2013; Nitschke & Schmieder, 2021).

A crucial element of polyglucan phosphorylation is its reversibility by the action of specific α-glucan phosphatases, since the presence of phosphoryl groups impedes the efficient mobilization of α-glucan chains by processive *endo*-acting enzyme classes such as β-amylase (BAM, EC 3.2.1.2) and starch phosphorylase (Pho1, EC 2.4.1.1) during starch degradation, and GP and GDBE during glycogen mobilization (Emanuelle, Brewer, Meekins, & Gentry, 2016; Frennett et al., 2022; Gentry, Brewer, & Vander Kooi, 2016; Irimia et al., 2015; Meekins, Vander Kooi, & Gentry, 2016). All known α-glucan phosphatases are members of the dual-specificity phosphatase (DSP, EC 3.1.3.48) family of proteins, and part of a larger protein tyrosine phosphatase superfamily (Fordham-Skelton et al., 2002; Kerk et al., 2006; Niittylä et al., 2006). In plants this includes Starch Excess 4 (SEX4) which removes C6 and C3 phosphate (Hejazi, Fettke, Kötting, Zeeman, & Steup, 2010; Hejazi, Fettke, Paris, & Steup, 2009), Like Sex Four2 (LSF2) which specifically removes C3 phosphates (Santelia et al., 2011), and LSF1 which is a catalytically inactive regulatory protein that stabilizes the amylopectin complex for amylolytic attack (Schreier et al., 2019). A recent study by Frennett et al. (2022) suggests that differences in kinetic properties between these enzymes in distinct plant tissues may account for observed variations in starch phosphate content. Loss of α-glucan phosphatase activity in plants leads to inefficient starch mobilization, excess starch in leaves at the end of the night, or inability to mobilize starch reserves in pollen, resulting in compromised growth and development (Kötting et al., 2009; Nashilevitz, Melamed-Bessudo, Aharoni, Kossmann, & Levy, 2009; Zeeman & ap Rees, 1999).

In plant starches the biological function of glucosyl phosphorylation appears to be closely related to turnover of the polymer, acting through electrostatic repulsion and the hydrophilic properties of the phosphate ester. Starches which undergo regular (diurnal) turnover, *e.g.* leaf starches (B-allomorph types) have relatively high levels of phosphate, whereas long-term storage starches (A-allomorph types) have low to undetectable phosphate (Blennow & Engelsen, 2010; Blennow, Engelsen, Munck, & Møller, 2000). Phosphorylation of starch by GWD and PWD occurs during starch biosynthesis (Castellanos, Khan, & Fettke, 2023; Nielsen, Wischmann, Enevoldsen, & Møller, 1994; Skeffington, Graf, Duxbury, Gruissem, & Smith, 2014; Xu et al., 2017) and the incorporated C6 and C3 phosphate groups disrupt glucosyl-glucosyl interactions. Specifically, C6 phosphate disrupts helix-helix interactions and C3 phosphate can unwind the α-glucan double helices (Blennow & Engelsen, 2010; Hansen et al., 2009) resulting in more open, hydrated structures which are amenable to hydrolytic amylases (Glaring et al., 2007; Mahlow et al., 2014). Introduction of C6 phosphate groups at the surface of amylopectin in starch granules enhances the binding and catalytic activity of BAM, a key enzyme class involved in starch degradation (Dudkiewicz, Simińska, Pawlowski, & Orzechowski, 2008; Edner et al., 2007; Frennett et al., 2022; Monroe, 2020). Phosphorylation of amylopectin at the surface of the starch granule by GWD and PWD probably primes the polymer for degradation *via* amylolysis (Hejazi, Mahlow, & Fettke, 2014; Mahlow et al., 2014).

The precise biological function of glycogen phosphorylation is not entirely clear. The presence of phosphate in normal glycogen may prevent insolubility of the particle as charged phosphoryl groups maintain water-solubility by keeping α-glucan chains and hydrophobic regions of the glycogen particle exposed to the aqueous milieu (Turnbull et al., 2013; Gentry, Guinovart, Minassian, Roach, & Serratosa, 2018; Nitschke et al., 2017; Sullivan, Minassian, & Nitschke, 2021). Lomako et al. (1994) proposed that phosphate acts as a “quality control” signal, based on observations that as phosphate accumulates the glycogen particle becomes less stable, less water-soluble, and more prone to be targeted for lysosomal degradation (Wisselaar, Kroos, Hermans, van Beeumen, & Reuser, 1993). Under normal conditions glycogen phosphate levels are regulated by the laforin phosphatase which is, in turn, regulated by AMP-activated protein kinase (Romá-Mateo et al., 2011). In tissues with relatively high glycogen phosphate contents such as skeletal muscle (Lomako et al., 1994; Nitschke & Schmieder, 2021) addition of phosphate may act as a control on the rate of degradation *via* GP and GDBE, ensuring significant amounts of muscle glycogen remain even after strenuous exercise for rapid resynthesis (Brooks & Gaesser, 1980; Prats et al., 2018). Phosphorylated glycogen is a better substrate for GS (Lomako et al., 1994) and may explain the observed rapid resynthesis of glycogen in muscle tissue following exhaustive exercise (Gaesser & Brooks, 1980; Kochan et al., 1979). All three proposed biological functions for glycogen phosphorylation are not mutually exclusive. The close structure/function and evolutionary relationships between starch and glycogen phosphatases are exemplified by the fact that human laforin is capable of dephosphorylating plant starch *in vivo* (Gentry et al., 2007).

The α-glucan phosphatase responsible for dephosphorylating glycogen is called laforin, named after its mutation, and is a major factor in Lafora disease (LD) (Gentry et al., 2018; Gentry & Pace, 2009; Worby, Gentry, & Dixon, 2006). LD is a progressive neurodegenerative myoclonus epilepsy caused by mutations in either laforin (*EPM2A*) or malin (*EPM2B*) (Andrade, Turnbull, & Minassian, 2007). Normal soluble glycogen formation requires that laforin and malin form a complex which is trafficked to the glycogen particle *via* laforin’s carbohydrate binding domain (CBM20) (Mitra et al., 2023; Skurat et al., 2024). LD is characterized by the formation of water-insoluble glycogen particles, termed polyglucosan bodies (PB), which show hyperphosphorylation and long α-glucan chains with reduced branching (Nitschke et al., 2013; Turnbull et al., 2010). Hyperphosphorylation of glycogen from *Epm2a^−/−^* and *Epm2b^−/−^* tissue appears to be associated with an altered water-insoluble α-glucan structure, *i.e.*, longer, unbranched chains. However, the fact that not all insoluble glycogen is hyperphosphorylated (*e.g.* in GBE1-deficiency), and that hyperphosphorylation *per se* does not cause glycogen insolubility speaks against hyperphosphorylation as a direct cause of the PB-driven pathology (Gayarre et al., 2014; Nitschke et al., 2017; Sullivan et al., 2019). The longer unbranched chains found in *Epm2a^−/−^* and *Epm2b^−/−^* polyglucosan bodies are thought to self-assemble in a similar manner to those of water-insoluble starches found in plants. Interestingly, the inverse relationship between α-glucan branching and phosphate deposition in LD glycogen has also been observed with plant starches. There is a strong correlation between unit chain length (CL) of amylopectin and degree of α-glucan phosphorylation, *i.e.*, starches with high levels of phosphate tend to have longer glucan chains and reduced branching frequency (Blennow, Bay-Smidt, Olsen, & Møller, 2000; Blennow, Bay-Smidt, Wischmann, Olsen, & Møller, 1998). Reducing expression of SBE genes in potato (*Solanum tuberosum* L.) and canola (*Brassica napus* L.) causes corresponding increases in glucan phosphate content in the starch of tubers and leaves, respectively (Wang et al., 2022; Wischmann et al., 2005) illustrating the strong negative correlation between glucan branching and phosphate content. In Arabidopsis (*Arabidopsis thaliana* L.) expression of the maize endosperm-specific SBEI which typically produces fewer branch points with longer branch chain lengths, resulted in leaf starch with approximately seven- to ten-fold higher α-glucan phosphate content than wild-type starch (Liu et al., 2016). The inverse relationship between branching of α-polyglucans by branching enzymes (BE) and α-glucan phosphate content of the polymer is the subject of analysis in the current work. Specifically, we aimed to test the hypothesis that the action of BEs is inhibited by the presence of α-glucan phosphates in glycogen and starch.

## 2. Materials and methods

### 2.1. Substrate preparation and dephosphorylation of α-glucan substrates

#### 2.1.1. Starch isolation

Maize endosperm starch was isolated from amyloplasts prepared according to methods described by Liu et al. (2009). Starch granules from plastid lysates were resuspended in cold aqueous washing buffer (50 mM tris (hydroxymethyl)-aminomethane (Tris)-acetate, pH 7.5, 1 mM Na2-EDTA, and 1 mM dithiothreitol [DTT]) and centrifuged at 3000 x*g* for 1 min at 4 °C. This washing step was repeated five times. The pellet was then washed three times with acetone followed by three washes with 2 % (*w*/*v*) sodium dodecyl sulfate (SDS). *Amylose extender* (*ae*) starches (*ae1.1* and *ae1.2* allelic variants) were extracted from the endosperm of dried kernels and washed as described above. Turmeric (*Curcuma longa* L.) starch was extracted from fresh rhizomes purchased from a local market according to methods described by Nakkala, Godiyal, and Laddha (2020). Potato amylopectin was purchased from Sigma-Aldrich (CAT#A8515) and used for dephosphorylation experiments as described below.

#### 2.1.2. Glycogen isolation

Rabbit (*Oryctolagus cuniculus* L.) liver glycogen (CAT#G8876) and oyster (*Ostreidae sp.*) glycogen (CAT#G8751) were obtained from Sigma-Aldrich. 100 mg of glycogen from Sigma was solubilized in 1 mL of dH_2_O at room temperature, then precipitated with 2.5 volumes of 95 % (*v/v*) ethanol with 15 mM LiCl at −20 °C for at least 1 h, followed by centrifuging for 5 min (4 °C, 16,000 x*g*). The supernatant from the centrifuge step was discarded, and the water solubilization and cold ethanol precipitation steps were repeated twice. Washed glycogen pellets were dried in a flow hood overnight, then dissolved in 900 μL of dH_2_O.

#### 2.1.3. Phytoglycogen isolation

Maize phytoglycogen (*sugary-1* mutant) was extracted and purified according to Bayliss, Shelton, Grossutti, and Dutcher (2021).

#### 2.1.4. Dephosphorylation of α-glucan substrates

Solubilized polyglucans were dephosphorylated by incubation with 5 units (U) (1 U defined as the amount of enzyme capable of dephosphorylating 1 μg of substrate into product per 10 min) of thermosensitive alkaline phosphatase (EC 3.1.3.1) (FastAP, ThermoFisher CAT#EF0651) per 10 mg of α-glucan in a buffer containing 10 mM Tris-HCl (pH 8.0), 5 mM MgCl_2_, and 100 mM KCl on a rotator at 37 °C for up to 24 h in order to hydrolyze all α-glucan phosphate monoesters. Efficiency and kinetics of the dephosphorylation reaction was monitored by measuring the release of inorganic phosphate from the α-glucan substrate over time.

### 2.2. Glucan phosphate determination

#### 2.2.1. Acid ammonium molybdate assay

Inorganic phosphate was measured using methods described by Taussky and Shorr (1953) using a KH_2_PO_4_ standard curve.

#### 2.2.2. Enzyme cycling assay

Glucan-bound phosphate was also quantified by a glucose 6-phosphate dehydrogenase (G6PDH, EC 1.1.1.49) redox-coupling assay performed as described by Nitschke et al. (2017) as a reference control compared to the acid ammonium molybdate assay.

### 2.3. Expression of recombinant ScGLC3 and DrGBE in E. coli

Recombinant BEs from brewer’s yeast (*Saccharomyces cerevisiae* L.) strain BY4742 (*Sc*GLC3) and *Deinococcus radiodurans* (*Dr*GBE) were cloned in the pET28a(+) plasmid (Novagen). The complete amino acid sequence of *Sc*GLC3 (GenBank accession no. BK006939.2) and *Dr*GBE (GenBank accession unavailable) were each constructed in a pET28a(+) backbone, *Sc*GLC3 with an N-terminal 6× Histidine tag and *Dr*GBE with a N-terminal 6× Histidine tag. The recombinant *Sc*GLC3 and *Dr*GBE were individually transformed to *E. coli* strains ArcticExpress (DE3) and BL21-CodonPlus (DE3)-RP, respectively. A single colony was inoculated in LuriaBertani (LB) media and grown overnight (37 °C, 250 RPM). Overnight culture was diluted 100 times and grown at 37 °C until an OD_600_ of 0.6–0.8 was reached. The expression of each recombinant protein was induced by addition of isopropyl β-D-1 thiogalactopyranoside (IPTG) to a final concentration of 1 mM (*Sc*GLC3) or 0.4 mM (*Dr*GBE) and the cultures were grown at 20 °C overnight (*Sc*GLC3) or 3 h at 34 °C (*Dr*GBE)*. E. coli* cells were collected by centrifugation at 3000 x*g* and approximately 1 mL of cell lysis solution (50 mM HEPES, 150 mM NaCl, 7.5 mM MgCl_2_, 0.1 % (*v*/v) Triton X-100, 0.4 mg lysozyme (BioBasic CAT#LDB0308), 1× ProteaseArrest (GBiosciences CAT#786–437)) was added per 50 mg of wet culture pellet. The pellet with cell lysis solution was resuspended by vortex and incubated on ice for 30 min. 10 U of DNaseI (ThermoFisher CAT#EN0521) per mL of lysis solution was added and incubated for 3 min at 37 °C, followed by 20 min on a rotator (at room temperature). The soluble protein fraction was separated from insoluble cell debris by centrifuging at 16,000 x g at 4 °C and the supernatant used for further protein purification. Recombinant proteins were purified using Poly-Prep Chromatography Columns (Bio-Rad CAT#7311550) and PureCube 100 Ni-NTA Agarose beads (Cube Biotech CAT#74103), following the manufacturer’s instructions. Protein purity was confirmed by SDS-polyacrylamide gel electrophoresis (PAGE) and western blotting.

### 2.4. Expression of recombinant human (Homo sapiens L.) HsGBE1 in HEK293T cells

A plasmid backbone was purchased from Vectorbuilder containing the following elements: ampicillin resistance, expression cassette 1 (CBh promotor, coding sequence 1, SV40 late pA), expression cassette 2 (CMV promotor, coding sequence 2 [mCherry-T2A-Puromycin resistance], SV40 early pA), SV40 origin of replication. A plasmid was generated containing the human *gbe1* sequence with an N-terminal 6 X Histidine tag. Plasmids were purified from Stellar chemically competent *E. coli* (Takara CAT#636763) using Qiagen QIAprep Spin Miniprep Kit (CAT#27104) according to manufacturer’s instructions prior to confirmation of sequence by whole plasmid sequencing (Plasmidsaurus). Plasmids were transfected into HEK293T (ATCC CAT#CRL-3216) cultured in DMEM (VWR CAT# 16750–072) with 10 % FBS (Fisher Scientific CAT#MT35010CV) using Lipofectamine 3000 (ThermoFisher CAT#L3000008) according to manufacturer’s instructions. Transfection was confirmed after 24 h by monitoring red fluorescence of reporter mCherry. From 48 h after transfection, cells were under continuous selection by 8 μg/mL puromycin (Gibco CAT#A1113803) with medium and antibiotic exchange every 48 h. Ten days after transfection, cells were frozen in aliquots and lysates prepared in RIPA buffer (50 mM Tris-HCl [pH 8], 150 mM NaCl, 1 % (v/v) Nonidet P-40, 0.5 % (*w*/*v*) sodium deoxycholate, 0.1 % (w/v) SDS). *Hs*GBE1 expression was confirmed by western blot following SDS-PAGE of 9 μg soluble cell lysate protein.

### 2.5. Preparation of plant cell extracts

Maize amyloplast extracts were prepared according to Liu et al. (2009) and used for western blotting. Potato tuber cell extracts were prepared by grinding potato tuber in liquid nitrogen, then subsequently ground in extraction buffer (100 mM Tricine [pH 7.8], 1 mM CaCl_2_, 5 mM MgCl_2_, 1 mM DTT) with 1× ProteaseArrest (G-Biosciences CAT#786–108). Homogenized extracts were centrifuged at 15,000 ×*g* (4 °C), the supernatant was taken for Bradford assay and subsequent gel affinity electrophoresis experiments.

### 2.6. Protein determination

Protein was measured using the Bio-Rad (Bradford) protein assay (Bio-Rad Laboratories Canada) according to the manufacturer’s instructions, using bovine serum albumin (BSA, Cytiva CAT#SH30574.02) as a standard.

### 2.7. BE activity assays

Catalytic activity of purified recombinant BEs was confirmed using the semi-quantitative iodine binding assay described by Borovsky, Smith, and Whelan (1975). BE activity was assayed by measuring *de novo* incorporation of [U-^14^C]-Glc-1-phosphate (Glc1P) into glucan chains by phosphorylase *a* using a modified version of a semi-quantitative assay described by Smith (1990).

### 2.8. Substrate affinity assay

Affinity electrophoresis was used as a means of measuring protein-glucan interactions, and *K*_d_ calculated from the retardation or relative electrophoretic mobility (*R*_m_) of the BE as affected by the α-glucan substrate contained in the polyacrylamide gel. We used methods described by Commuri and Keeling (2001) and Matsumoto, Nakajima, and Matsuda (1990). A two-sample *t*-test for independent samples was used to determine the statistical significance of enzyme mobility with or without glucan-bound phosphate, and linear regression was conducted using GraphPad. All statistical analyses were conducted with a confidence interval of 95 %.

### 2.9. Preparation and analysis of polyclonal antibodies and western blotting

Polyclonal rabbit antisera targeted to maize SBEIIa or SBEIIb, and potato SBEI were raised against synthetic peptides prepared as previously described (Liu et al., 2009), and for yeast *Sc*GLC3, PNVNNGDSYKYARRQC, corresponding to amino acid residues 557–572 of the full-length protein (GenBank acc. no. AAA34632). Purified monoclonal antibody for human GBE1 (*Hs*GBE1) was obtained from Abcam (CAT#ab180596) raised in rabbit. Non-denaturing gels were blotted onto nitrocellulose membrane (BioRad CAT#1620115) and blocked in 1.5 % (w/v) BSA using the methods described by Harlow and Lane (1999).

### 2.10. Determination of CL distribution of polyglucans

The CL distribution (CLD) of debranched polygucans was determined following separation on a Dionex ICS 3000 high-performance anion-exchange chromatography with pulsed amperometric detection (HPAEC-PAD) system with a CarboPac PA-100 ion-exchange column (4 × 250 mm) and guard column (4 × 50 mm). Eluents and elution method used was as described by O’Shea and Morel (1996) and Bertoft (2004). To determine the CLD of each sample, the average CL was calculated following the methods of Annor, Marcone, Bertoft, and Seetharaman (2014) and Bertoft, Piyachomkwan, Chatakanonda, and Sriroth (2008).

## 3. Results

### 3.1. Glucan phosphate determination in starches and glycogens from a range of biological sources

Glucan-bound phosphate was measured in a range of α-polyglucans from different biological sources using two distinct analytical methods. We employed a previously optimized enzymatic cycling assay for analysis of Glc6P which has sufficient sensitivity for the accurate analysis of low levels of glucan phosphate (μg- to mg-levels) of α-polyglucans (Nitschke et al., 2013). For routine analysis of covalently bound phosphate from starch (amylopectin) we measured free inorganic phosphate released following treatment of polyglucans with alkaline phosphatase (FastAP) using a colorimetric method (Taussky & Shorr, 1953). Comparison of the two methods using potato amylopectin indicates that they are equivalent in terms of their accuracy in determining α-glucan-bound phosphate levels (Fig. S1).

**Fig. 1.**
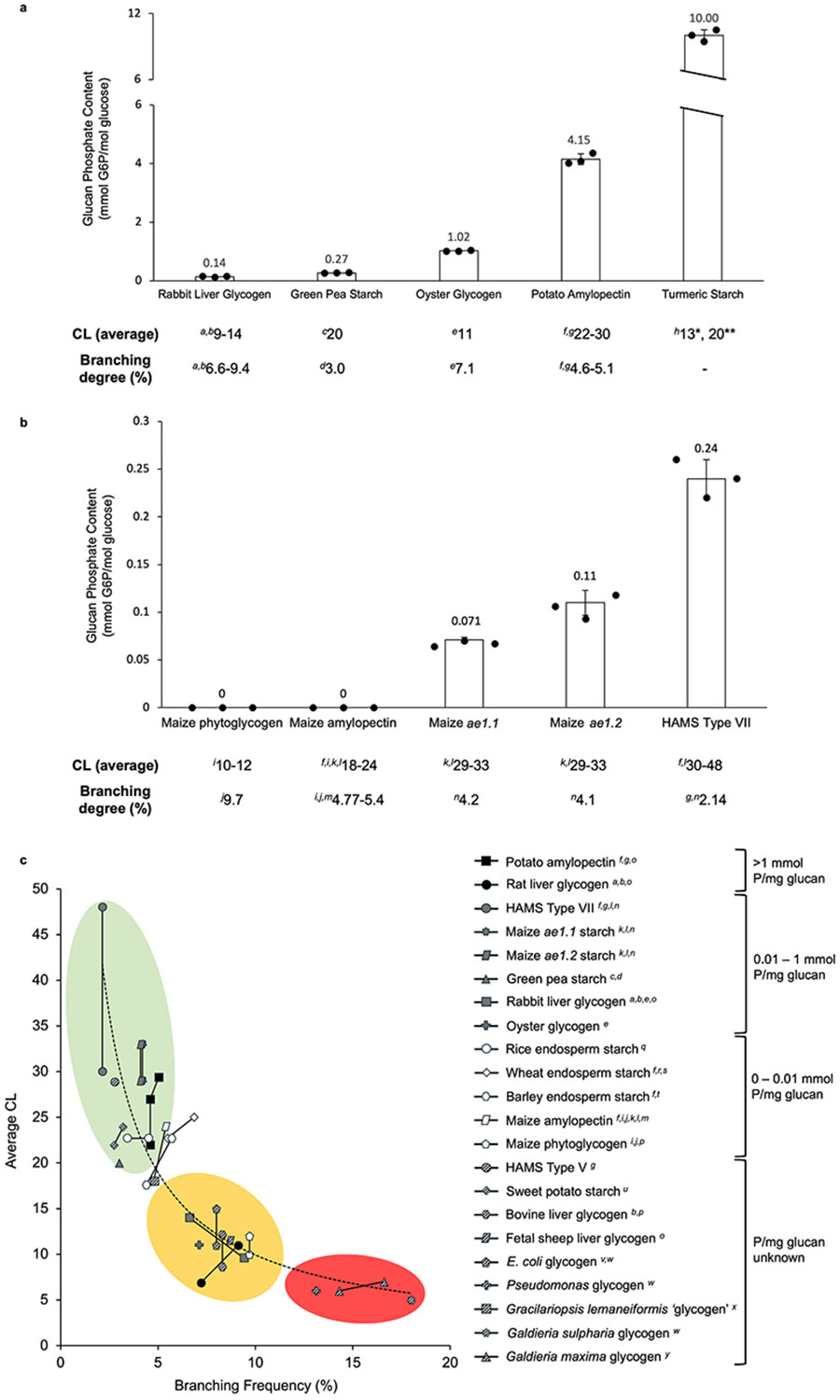
Phosphate contents of α-polyglucans from different biological sources. Approximately 0.5–1.0 mg gelatinized starch and washed glycogen was used to determine glucan phosphate using a G6PDH-linked enzyme cycling assay. (a) Plant starches from various botanical origins and organs, and different biological sources of glycogen. (b) Maize endosperm starch phosphate from genetic backgrounds with varied branching enzyme activity. Phytoglycogen is a result of the *sugary-1* mutation which causes an elevated branching frequency in the polyglucan compared with normal amylopectin. Data presented represents the mean ± standard deviation (S.D.) of three replicates. This data shows an inverse relationship between starch phosphate and amylopectin branching frequency, and a positive correlation between glucan phosphate and polymer average chain length (CL). The tables below each histogram summarize key structural characteristics of the corresponding polyglucan and are derived from previously published data. The – symbol signifies no available information, asterisks identify turmeric starch from different species: * turmeric (*Curcuma longa* L.), **black turmeric (*Curcuma caesia* L.). ^*a*^Tolmasky and Krisman (1987), ^*b*^Ryu et al. (2009), ^*c*^Chung and Liu (2012), ^*d*^Gaenssle, Satyawan, Xiang, and van der Maarel (2021), ^*e*^Akai, Yokobayashi, Misaki, and Harada (1971), ^*f*^Jane et al. (1999), ^*g*^Martinez et al. (2018), ^*h*^Hung and Vo (2017), ^*i*^Inouchi et al. (1987), ^*j*^Pan et al. (2023), ^*k*^Yun and Matheson (1993), ^*l*^Klucinec and Thompson (2002), ^*m*^Seo et al. (2002), ^*n*^Liu et al. (2012). (c) Relationship between average CL and α-1,6-branching frequency in a range of α-polyglucans with approximate α-glucan phosphate levels denoted by icon colour. Black icons for high levels of phosphate (>1 mmol P/mg glucan), dark grey icons for medium levels of phosphate (0.01–1 mmol P/mg glucan), white icons for undetectable or trace levels of glucan phosphate (0–0.01 mmol P/mg glucan), and patterned icons for unknown levels of glucan phosphate. Superimposed over the curve are coloured regions which depict trends in glucan phosphate content based on average CL and branching frequency of the polyglucan; green represents high, yellow for medium to low, and red for undetectable α-glucan phosphate. ^*o*^Illingworth, Larner, and Cori (1952), ^*p*^Matsui, Kakuta, and Misaki (1993), ^*q*^Van Leeuwen et al. (2021), ^*r*^Liu, Qiao, Kong, Wang, and Yang (2024), ^*s*^Zhang et al. (2017), ^*t*^Li et al. (2024), ^*u*^Guo et al. (2022), ^*v*^Wang et al. (2019), ^*w*^Martinez-Garcia, Stuart, and van der Maarel (2016), ^*x*^Yu et al. (2002), ^*y*^Stadnichuk, Semenova, Smirnova, and Usov (2007).

We observed wide variation in the amount of bound phosphate in starch and glycogen from various biological sources (Fig. 1a). Starch extracted from rhizomatous and tuberous plant tissues (B-type allomorph) showed highest levels of phosphate (10.0, and 4.12 μmol/mg glucan in turmeric rhizome and potato tuber, respectively) in agreement with previous studies (Blennow, Bay-Smidt, et al., 2000), whilst storage starches from cereal endosperms (A-type allomorph) show barely detectable levels of α-glucan-bound phosphate (0.0004 mmol P/mol Glc in rice endosperm; Jane et al., 1999) which is in accordance with previously published measurements (Blennow, Engelsen, et al., 2000).

Wide variation was observed among the phosphate contents of glycogen particles (Fig. 1a), with rabbit liver glycogen phosphate levels (0.14 mmol/mol Glc) comparable with some plant storage starches, and oyster glycogen phosphate 7-fold higher than that of rabbit liver glycogen.

We could not detect any starch phosphate in the storage starch of maize endosperm (Fig. 1b) which agrees with previous findings (Blennow, Engelsen, et al., 2000). The maize *amylose extender* (*ae*) mutant is caused by loss of *Zm*SBEIIb, the major starch branching enzyme activity in the endosperm, resulting in altered granule morphology, amylopectin with reduced branching frequency, fewer short α-glucan chains and a higher apparent amylose content (increased long amylopectin chains) (Klucinec & Thompson, 2002). Allelic variants of *ae* maize (termed *ae1.1* and *ae1.2*) show varied amylopectin branching frequency (Liu et al., 2012). Commercial high amylose maize starches (HAMS, TypeV and VII) represent different forms of *ae* starch (Jiang et al., 2010). Structural analyses of normal maize endosperm starch, phytoglycogen and *ae* starches are summarized in Fig. 1b. It is noteworthy that as polyglucans become less branched (% degree of branching), average CL increases and α-glucan phosphate increases in the different maize genotypes. Fig. 1b shows a negative correlation between branching frequency (modulated by reduced SBE gene expression and SBE activity) and amylopectin phosphate content, *i.e.*, as BE activity is suppressed, more long (unbranched) α-glucan chains are produced, branching frequency is reduced, and α-glucan phosphate content increases. Water-soluble phytoglycogen produced in the *sugary-1* maize endosperm is a result of suppressed debranching activity by isoamylase-1, resulting in a higher branching frequency (approximately 9.7 %) compared with normal amylopectin (approximately 5.4 %, Inouchi et al., 1987). Interestingly, there was no detectable phosphate measured in maize phytoglycogen (Fig. 1b). The lack of phosphate in phytoglycogen is probably of no functional significance, but rather a reflection of the fact that the high frequency of short, open, soluble α-glucan chains in the polymer are a poor substrate for GWD and PWD (Hejazi et al., 2008).

Fig. 1c shows the relationship between average CL and branching frequency (% α-1,6-branch linkages) in a range of starches and glycogens from various biological sources. Within the physicochemical restrictions on the limits of polyglucan branching, *i.e.*, between 2.14 % (HAMS, typeVII; Martinez et al., 2018) and 18 % (*Galdieria sulphuraria* [red algal] glycogen, Martinez-Garcia et al., 2016) it appears that as branching frequency increases, average CL decreases. Water-insoluble plant starches have relatively low branching frequencies (≤ 5.4 %) and longer average CLs (Fig. 1c). Suppression of SBE genes in plants (*e.g. ae* and HAMS) reduces branching and results in longer average CLs compared with normal starches. Starches with longer average CLs show higher levels of glucan phosphate compared with polyglucans with short average CL (*e.g.*, glycogen) (Fig. 1a-c).

### 3.2. Substrate affinity of yeast glycogen branching enzyme (ScGLC3) is reduced by α-glucan phosphorylation

**Fig. 2.**
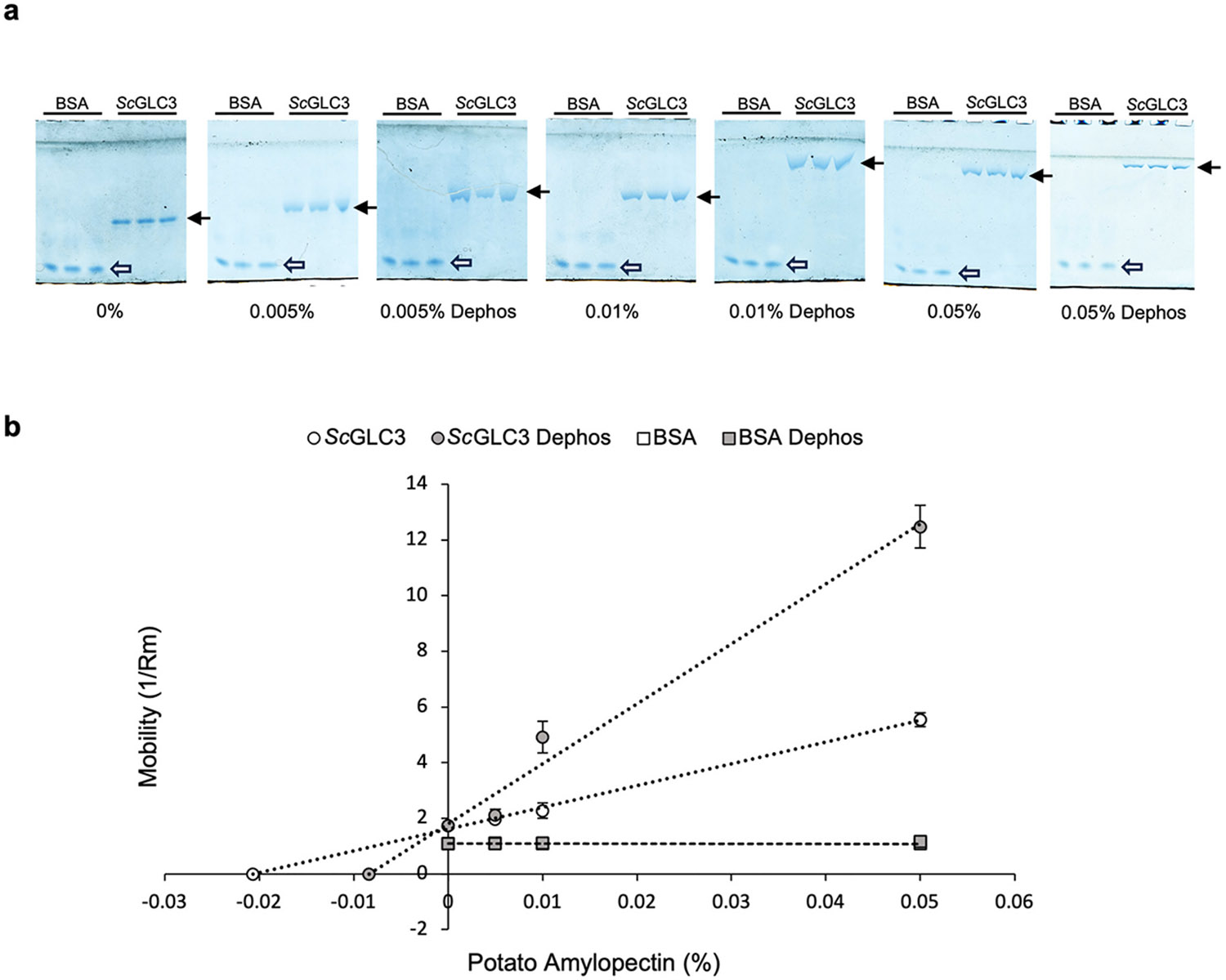
Determination of BE mobility by affinity gel electrophoresis. Data shown is for yeast *Sc*GLC3, identical methods were used for subsequent analysis of BEs from other biological sources. (a) Recombinant *Sc*GLC3 (5 μg per lane) was separated on non-denaturing 5.6 % (*w*/*v*) polyacrylamide gels containing various concentrations of untreated potato tuber amylopectin (~4 mmol Glc6P/mg Glc) or dephosphorylated (dephos) potato tuber amylopectin. Gels were visualized using Coomassie Blue R-250 staining. Mobility of *Sc*GLC3 (black arrow) is reduced with increasing amylopectin concentrations, and further reduced in dephosphorylated amylopectin gels. The mobility of BSA (white arrow) is unaffected by the presence of glucan (phosphorylated or dephosphorylated) in the gel matrix. (b) Plots of the reciprocal of relative mobility (1/*R*_m_) of *Sc*GLC3 and BSA against the concentration of untreated potato amylopectin (white symbols) and dephosphorylated potato amylopectin (grey symbols). BSA mobility in dephosphorylated potato amylopectin is superimposed over BSA mobility in untreated potato amylopectin. Relative affinity (1/*K*_d_) of *Sc*GLC3 for α-glucan substrates is determined from the inverse of the *x*-intercept of the plot and these values, together with 1/*K*_d_ measurements derived for other BEs are given in subsequent figures. Data is the mean ± standard deviation (S.D.) of 3–5 independent experiments.

We employed affinity gel electrophoresis to analyze the affinity of BEs for phosphorylated α-glucans. The dissociation constant (*K*_d_) of the GBE from the ascomycete brewer’s yeast (*Saccharomyces cerevisiae* L.) (*Sc*GLC3) was determined by measuring the relative migration (*R*_m_) of recombinant *Sc*GLC3 in the presence of phosphorylated and dephosphorylated α-glucan substrates (Fig. 2a). Reciprocal values of *R*_m_ (1/ *R*_m_) of *Sc*GLC3 are linearly related to the concentration of α-glucan substrate in the gels, and *K*_d_ is measured as the intercept on the *x*-axis of this relationship (Fig. 2b). Substrate affinity is given as 1/*K*_d_ (Matsumoto et al., 1990). We used bovine serum albumin (BSA) as a control protein in all affinity gel electrophoresis experiments. BSA showed no alteration in migration, irrespective of glucan, glucan concentration, or phosphate content of glucan (Fig. 2a). The mobility behaviour of *Sc*GLC3, and other BEs (see below), in α-glucan-containing gels and in relation to the BSA control indicates that their mobility is a function of their specific affinity for particular α-glucan substrates. The affinity of *Sc*GLC3 for potato amylopectin was increased approximately 2.5-fold following removal of phosphate by FastAP (Fig. 2b).

### 3.3. BEs from a range of biological sources show reduced substrate affinity with phosphorylated α-glucan substrates

**Fig. 3.**
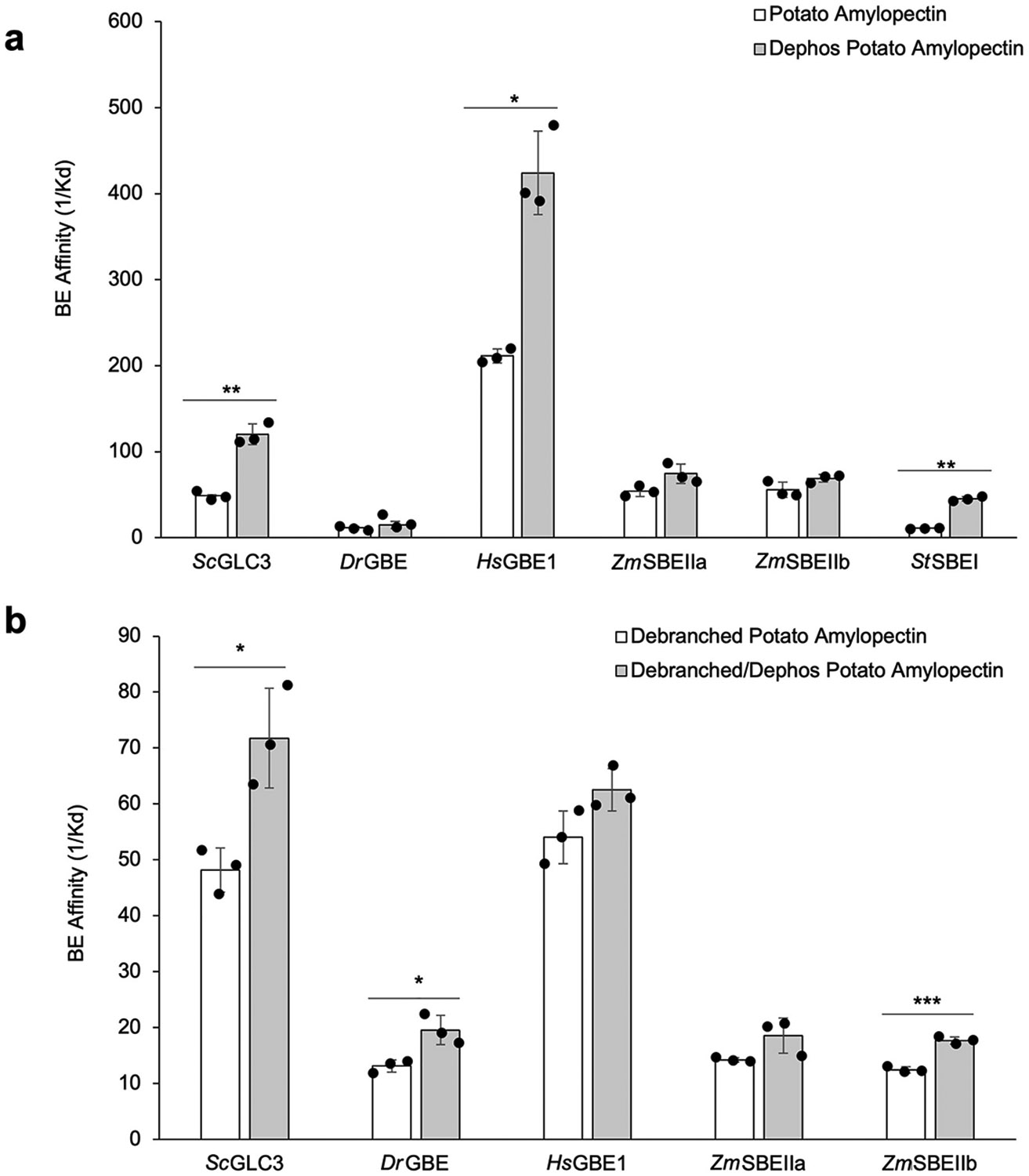
Relative substrate affinity (1/*K*_d_) of different BEs for (a) phosphorylated and dephosphorylated potato tuber amylopectin, and (b) phosphorylated and dephosphorylated debranched amylopectin α-glucan chains (MOS). Untreated (phosphorylated) potato tuber amylopectin contained ~4 μmol P/mg glucan. Data presented is the mean ± S.D. of 3–5 independent experiments. *P < 0.05, **P < 0.01, ***P < 0.001.

Several starch and glycogen BEs were tested for the effects of glucan phosphorylation on their respective substrate affinities using potato tuber amylopectin. Potato tuber amylopectin was selected for these experiments because of its high phosphate content compared to other starches. Catalytically active recombinant GBEs from prokaryotic (bacterial) (*Deinococcus radiodurans*, *Dr*GBE) and eukaryotic sources (human (*Homo sapiens* L.), *Hs*GBE1) as well as SBEs from maize endosperm (*Zm*SBEIIa and *Zm*SBEIIb) and potato tuber (*St*SBEI) extracts were separated by affinity gel electrophoresis according to methods described above for *Sc*GLC3. Fig. 3a shows that, in common with the behaviour of yeast GBE *Sc*GLC3, the substrate affinity of other BEs for potato amylopectin is markedly increased following dephosphorylation of the substrate with FastAP, suggesting a general inhibitory effect of covalently-linked α-glucan phosphate residues on the substrate affinity of these enzymes. All BEs tested showed reduced mobility (*i.e.*, increased substrate affinity) in substrate gels containing dephosphorylated glucan compared with those containing phosphorylated glucan substrates, with *Sc*GLC3, *Hs*GBE1, and *St*SBEI showing significant increases in substrate affinity (Fig. 3a).

To determine whether the observed mobility changes of different BEs following the dephosphorylation of the α-glucan substrate is a direct effect of the presence of phosphate, or a consequence of alterations in the three-dimensional structure of the gelatinized amylopectin, *e.g.* increased local crystallinity and/or helix formation caused by dephosphorylation, we debranched potato tuber amylopectin with *Pseudomonas* isoamylase and used the resulting linear malto-oligosaccharides (MOS) as substrates in affinity gels. It can be seen from Fig. 3b that when debranched MOS are used as a substrate for BEs a similar inhibitory effect of glucan phosphate is observed to that of intact amylopectin (*c.f.* Fig. 3a). *Hs*GBE1 showed a markedly lower affinity (1/*K*_d_ value) with MOS than potato amylopectin, and the inhibitory effects of phosphate observed with amylopectin were not seen with MOS (Fig. 3b). All BEs showed reduced mobility after dephosphorylation of MOS, though only *Sc*GLC3, *Dr*GBE and *Zm*SBEIIb were found to be significant.

Similar substrate experiments with BEs were conducted using intact oyster glycogen which has relatively high phosphate content (Fig. S2). All BEs tested showed extremely low affinity for glycogen, irrespective of its phosphorylation state when compared with amylopectin substrates (Fig. 3). *Dr*GBE and *Zm*SBEIIa showed statistically significant increased substrate affinity following dephosphorylation of oyster glycogen. The likely explanation for the low substrate affinities of BEs for glycogen is the fact that intact “full-size” glycogen particles have a high frequency of branch points at their periphery (Pan et al., 2023) making further interaction and utilization by BEs difficult.

### 3.4. Affinity of BEs for potato amylopectin is directly related to α-glucan phosphate content

**Fig. 4.**
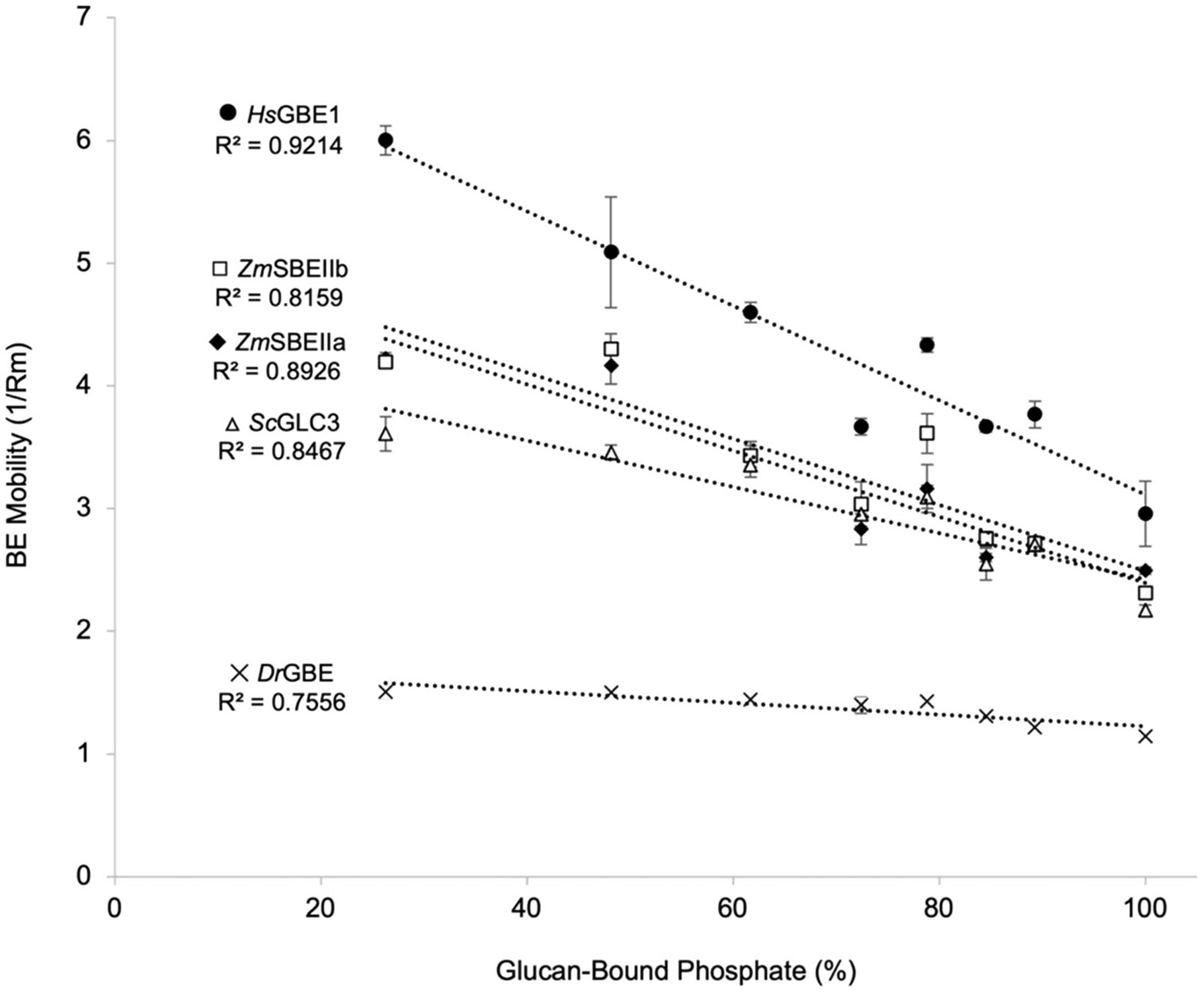
Titration of α-glucan phosphate content with mobility of various BEs separated by affinity gel electrophoresis. Mobility of various BEs (1/*R*_m_) was determined in gels containing potato amylopectin of various degrees of phosphorylation. Data shows a negative linear relationship between enzyme mobility through an α-glucan substrate and the degree of glucan phosphorylation for all BEs studied. Data presented represents the mean ± S.D. of 3 replicate experiments. The relative inhibitory effect of phosphate on affinity of the BEs for the substrate can be estimated by the slope of the line for each enzyme.

The substrate affinity of various BEs was tested over a range of amylopectin phosphate values. Reaction conditions were optimized to create a range of potato tuber amylopectin with different concentrations of phosphate by hydrolytic release using FastAP (Fig. S3). The mobility of various BEs in substrate affinity gels containing different levels of glucan phosphate was determined (Fig. 4 and Fig. S3). Results show a statistically significant linear relationship between the mobility of different BEs in gels containing gelatinized potato tuber amylopectin and the glucan phosphate content (Fig. 4). The *p*-values of the linear regressions of 1/*R*_m_ *versus* % glucan phosphate for the respective BEs was 0.0002 (*Hs*GBE1), 0.0021 (*Zm*SBEIIb), 0.0004 (*Zm*SBEIIa), 0.0012 (*Sc*GLC3), and 0.0051 (*Dr*GBE). As phosphate content increases to the maximum observed in normal potato amylopectin (100 %; ~4 mmol Glc6P/mol Glc) the mobility of the various BEs accordingly increases (*i.e.*, 1/*R*_m_ decreases, corresponding to a decrease in substrate affinity). The relative degree of sensitivity of BEs to glucan phosphate can be estimated from the slope of the linear relationship for each enzyme (Fig. 4, inset). *Hs*GBE showed the greatest sensitivity to α-glucan-bound phosphate in the amylopectin gels, with *Dr*GBE showing the least sensitivity out of the five enzymes tested.

### 3.5. Measuring the effects of α-glucan phosphate on catalytic activity of BEs

The catalytic activity of various BEs was determined by measuring changes in CLD of debranched polyglucan products following their incubation with phosphorylated or dephosphorylated substrates. We reasoned that this approach was a more direct and informative assay of BE action than other semi-quantitative techniques. Incubation of *Sc*GLC3 and *Dr*GBE with potato amylopectin produced different branched polyglucans (Fig. S4). The presence or absence of phosphate on the amylopectin substrate had no effect on the catalytic action of the BEs tested (Fig. S4). Both *Sc*GLC3 and *Dr*GBE showed branching of potato amylopectin, but analysis of the branching patterns of both enzymes showed no difference between the FastAP-treated potato amylopectin and the untreated control (Fig. S4 and Table S1 shows data for *Dr*GBE and *Sc*GLC3). We also employed the ^14^C-based phosphorylase *a*-stimulation assay as a measure of BE catalytic activity. This optimized assay showed no difference in catalytic activity of the BEs tested between phosphorylated and dephosphorylated amylopectin (Fig. S5a and b shows data for *Sc*GLC3).

## 4. Discussion

**Fig. 5.**
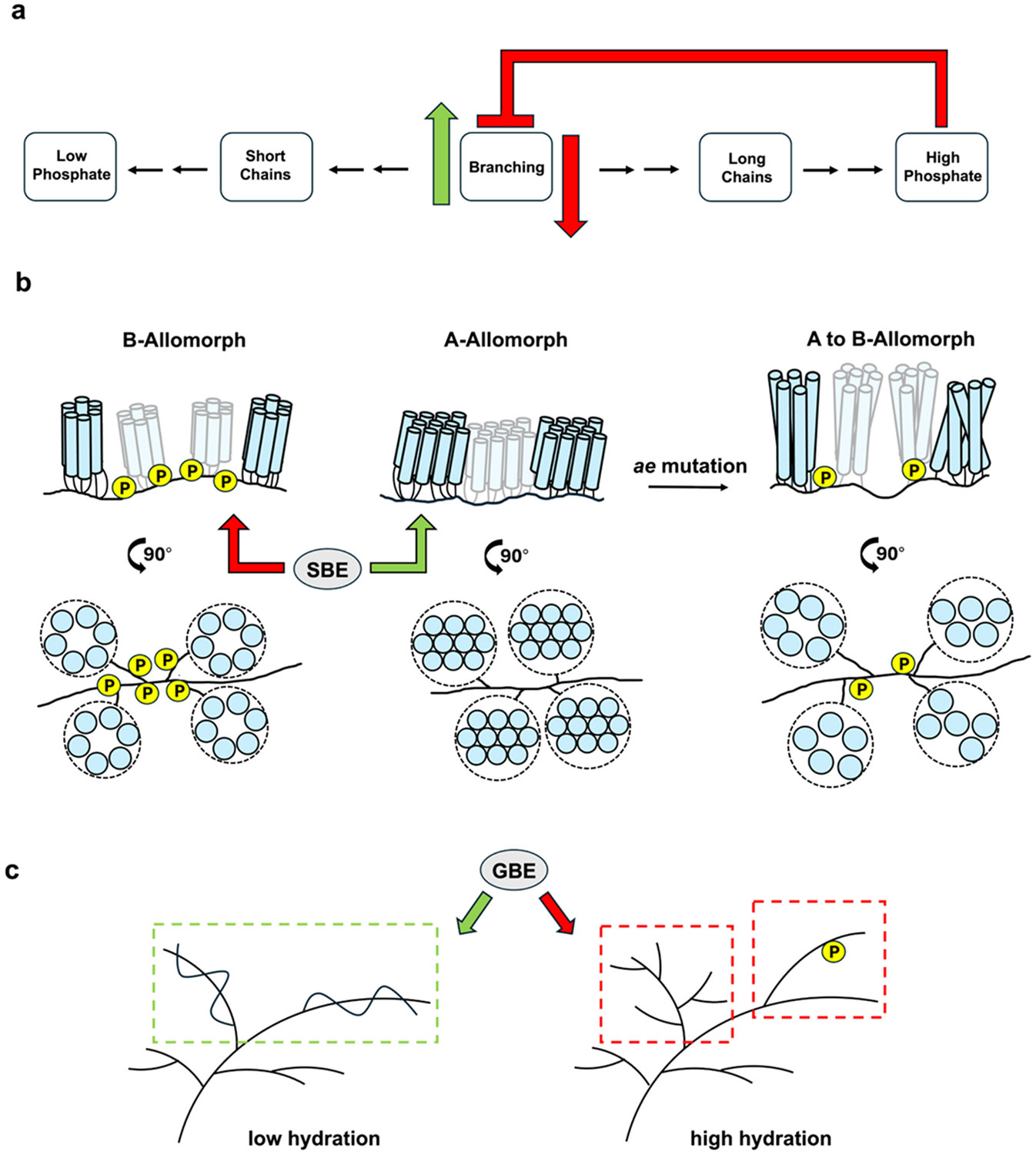
Proposed mechanistic models outlining the observed relationship between glucan phosphate and branching frequency in starch and glycogen. (a) Generalized schematic illustrating the effects of modulating BE activity (and therefore the branching: elongation ratio) in starch and glycogen. Increasing branching activity results in polymers with increased branching frequency, shorter average CL, and low glucan phosphate (*e.g.*, glycogen, phytoglycogen). Reduced BE activity reduces polymer branching frequency resulting in longer average CL and higher glucan phosphate. The current study suggests that increased glucan phosphate reduces the affinity of BEs for their substrates. (b) Ultrastructural characteristics of plant starches underpin their distinct biological functionalities. Transient starches (B-type allomorph) have relatively high phosphate content thought to be deposited in the long chains connecting cluster regions, making these regions (see 90° view) more hydrated. Storage starches (A-type allomorph) have low, or no measurable phosphate and have smaller inter-cluster spacing, creating more compact, less hydrated cluster regions. Reduced BE activity in normally A-type allomorph storage starches, *e.g.*, in maize *ae* mutants, causes structural alterations leading to a B-type allomorph with reduced branching frequency, increased average CL, and more open, hydrated clusters and increased glucan phosphate. Higher levels of glucan phosphorylation found in the longer cluster-connecting regions of the amorphous regions of B-allomorph amylopectin may create localized regions which inhibit branching, leading to more open, hydrated structures. c) Normal water-soluble glycogen (high hydration) is maintained both through branching and glucan phosphorylation (red boxes). Longer, unbranched and non-phosphorylated chains (green box) have low hydration, making the glycogen particle prone to instability, yet are optimal substrates for GBE (green arrow). The catalytic actions of GBE, and an unknown glycogen phosphorylating enzyme maintain hydration and stability of the complete glycogen particle, for which GBE has reduced affinity (red arrow).

Starch and glycogen are both phosphorylated to differing degrees, although the precise biological function of α-glucan phosphorylation likely differs between the two polyglucans. For example, in plants α-glucan phosphorylation serves an important role in priming the water-insoluble starch granules for degradation *via* hydrolytic enzymes such as BAM, whereas its role in the metabolism of water-soluble glycogen particles is less clear. Despite differences in structural organization and metabolism between starch and glycogen, clear patterns of phosphate deposition are observed in relation to common structural characteristics of both polymers, perhaps suggesting similar behavioural properties of the enzymes involved in α-glucan phosphorylation in relation to their respective α-glucan substrates. The model presented in Fig. 5 summarizes evidence from multiple studies, including this study, showing how modulation of BE substrate affinity, and therefore polymer branching degree, causes measurable changes in α-glucan phosphate levels in starch and glycogen. The observed patterns of α-glucan phosphate deposition in starch and glycogen appear to rest in part upon the structural relationship between average CL and branching degree (Fig. 1c), supporting many published observations showing that α-glucan phosphorylation tends to increase in polyglucans with reduced branching frequency and longer α-glucan chains (Blennow et al., 1998; Blennow, Bay-Smidt, et al., 2000; Blennow, Engelsen, et al., 2000; Sullivan et al., 2019, Wang et al., 2022; Samodien et al., 2018; Fig. 1c). The precise mechanism underpinning this relationship is not clear; for example, those polyglucans with the highest phosphate levels (*e.g.*, starches of tuberous and rhizomatous storage tissues) are amylopectins with only moderately higher average CL, and lower branching frequency compared with other sources of amylopectin (Fig. 1c). Genetic manipulation of average CL (and therefore branching frequency) *via* the suppression of BE gene expression stimulates α-glucan phosphate deposition in amylopectin, but only up to a certain degree (see Fig. 1b and c). For example, the α-glucan phosphate content of Type VII HAMS (which is a result of SBEIIb suppression) is >17-fold, and > 40-fold less than potato and turmeric starch, respectively, yet has relatively high average CL (DP 31–48) and very low branching frequency (~2.14 %, Fig. 1b) in comparison with these, and similar tuberous starches. Therefore, the factors responsible for the variation in α-glucan phosphate levels found in normal starches and glycogens are not fully understood.

In wild-type starches, phosphorylation of the amylopectin component is a product of the actions of GWD and PWD, and phospho-glucan turnover (dephosphorylation) by SEX4 and LSF2. A study by Frennett et al. (2022) showed there is wide variation in the catalytic activities of SEX4 orthologs from different plant species which may account for some of the variation in observed α-glucan phosphate levels of their respective starches. For example, cereal SEX4 from rice and maize endosperm (which make A-type allomorph storage starches with low, to no detectable α-glucan phosphate) showed higher rates of dephosphorylation than SEX4 orthologs from storage tubers such as potato and cassava (*Manihot esculenta* L.) (Frennett et al., 2022). Similarly, species-specific variations in gene expression, catalytic activities and properties of the α-glucan phosphorylating enzymes GWD and PWD may also influence native starch phosphate levels. *In vitro* studies with GWD from potato tuber suggest a preference for B-type allomorph starch (Fettke et al., 2009) and crystalline, rather than soluble, maltodextrins as a substrate (Hejazi et al., 2008; Hejazi et al., 2009). These data suggest that structural, ordered features of α-polyglucans are probably more important for the functioning of GWD than their chemical properties *per se.* GWD’s actions during starch biosynthesis or degradation may therefore be restricted to linear, unbranched α-glucan substrates with vicinal double helices. Further support for the proposed *in vivo* substrate preferences of GWD come from a number of detailed amylopectin fractionation studies aimed at elucidating the specific location of α-glucan phosphate residues in starch granules. Studies with potato tuber amylopectin indicates that α-glucan phosphates (particularly C6 phosphate) accumulate on long, linear cluster-connecting chains found in the so-called amorphous regions of the starch granule (Blennow, Bay-Smidt, et al., 2000; Wikman et al., 2013; Wikman, Larsen, Motawia, Blennow, & Bertoft, 2011), which is in turn consistent with the notion that α-glucan phosphate deposition occurs synchronously with amylopectin biosynthesis (Castellanos et al., 2023; Hejazi et al., 2014; Nielsen et al., 1994). Phosphorylation of amylopectin chains by GWD and PWD may therefore serve different, yet complementary, biological roles. On one hand, phosphate deposition during starch biosynthesis may facilitate aspects of fine structural assembly and organization, and on the other hand, the actions of GWD and PWD at the surface of the “finished” starch granule structure is an important mechanism by which the process of granule degradation by hydrolytic enzymes is primed, particularly during the diurnal turnover of leaf starch. A recent study by Compart, Apriyanto, and Fettke (2023) may support this proposed dual function of α-glucan phosphorylation in starch metabolism. Analysis of the products of recombinant potato and oil palm (*Elais guineensis* L.) GWDs on insoluble, pre-formed starch granules showed that GWD works on highly ordered (helical) chains at the granule surface *in vitro,* disrupting them through the phosphorylation of glucosyl units within the vicinity of α-1,6-branch points connecting vicinal glucan chains (Compart et al., 2023; Li et al., 2024). This data, showing glucan phosphate deposition in the vicinity of α-1,6-branch points, appears to run counter to the observed structural data outlined above, which shows that there is an inverse relationship between branching and α-glucan phosphate deposition, and that phosphate groups are found on longer α-glucan chains *in vivo*. However, these different findings are not necessarily mutually exclusive. The “finished” starch granule surface probably represents a distinct physicochemical milieu with respect to the nature of available α-glucan substrates, the general localized hydration state, and the presence of different enzymes and regulatory proteins compared with the corresponding micro-environment prevailing during assembly of the nascent granule; each micro-environment possibly influencing the catalytic actions of GWD and PWD in different ways. The general physicochemical consequences of α-glucan phosphorylation therefore appear to be the same, *i.e.*, the promotion of localized regions of hydration by opening vicinal α-glucan chains which are otherwise prone to self-assembly and insolubility. In plants this process may be important in 1) maintaining disordered, hydrated regions within the amorphous layers of the growing granule, and 2) in facilitating the process of starch degradation by disruption of tightly packed α-glucan chains on the surface of the granule.

In mammalian glycogen biosynthesis the stability (*i.e.*, water solubility) of the glycogen particle is key to the prevention of the formation of insoluble aggregates termed PB, causal agents of pathological states such as LD, Glycogen Storage Disease IV (GSD4, Anderson’s Disease), and polyglucosan body myopathy1 (Nitschke et al., 2022). However, the interrelationship between branching frequency, α-glucan phosphate and glycogen particle solubility is complex and unclear. For example, in mice GBE1 deficiency produces a water-insoluble fraction of muscle glycogen with lower branching frequency and longer average CL (comparable with *ae* mutants in plants, Fig. 1b), but no increase in α-glucan phosphorylation (Sullivan et al., 2019). Furthermore, laforin deficient mice over-expressing a laforin with a point mutation resulting in a catalytically inactive α-glucan phosphatase domain produce hyperphosphorylated glycogen which, unlike the hyperphosphorylated glycogen of LD, is water-soluble (Gayarre et al., 2014; Nitschke et al., 2017). These studies highlight the importance of the laforin-malin protein complex for preventing the synthesis of abnormal glycogen particles. Nevertheless, glycogen phosphate deposition, *via* a yet unidentified agent, may still be critical in maintaining solubility together with coordinated control of branching and elongation by GBE and GS, respectively. Not all glycogen-synthesizing organisms (*e.g.*, insects, yeasts and bacteria) have laforin (Gentry & Pace, 2009), and it is interesting to speculate whether the glycogen in these organisms is phosphorylated, and also to note that some, *e.g., Galdieria* and *Pseudomonas sp.*, have highly branched glycogen with very short CLs (Martinez-Garcia et al., 2016), perhaps obviating the requirement for phosphorylation as a mechanism for maintaining solubility of these glycogen particles. The short average CL of bacterial glycogen is associated with lower rates of polymer turnover and increased stress resistance and fitness (Wang & Wise, 2011; Wang et al., 2020).

In view of the close relationship between α-glucan branching and phosphate deposition in the polyglucans described above, this study aimed to examine the effects of extant α-glucan phosphorylation on the actions of BEs. Specifically, we wished to test the hypothesis that phosphorylation of α-polyglucans inhibits the subsequent actions of BEs. Affinity gel electrophoresis is an effective method for measuring substrate affinities of carbohydrate-active enzymes, including BEs (Commuri & Keeling, 2001; Matsumoto et al., 1990). Our data show that the presence of α-glucan phosphate in starch reduces the substrate affinity and binding of BEs. We tested a wide biological range of BE sources from prokaryotes (bacteria) to eukaryotes (plants, yeast, and human), and amylopectin interaction of all BEs tested were inhibited to varying degrees by covalently bound phosphate (Fig. 4), allowing us to suggest that this is a general phenomenon, perhaps relating to common features of the structure and function of the BE enzyme class. Starches have closely packed branches in the crystalline regions of the granule, making neighbouring chains likely to self-assemble and potentially create local structural environments that could impact BE function. For example, it has been noted that some SBEs show substrate preference for helical coils comprising closely associating α-glucan chains (Borovsky et al., 1975). Our experiments were conducted with gelatinized substrates, making it unlikely that the formation of such secondary structures is influencing the interpretation of these observations. Furthermore, experiments involving debranched substrates (Fig. 3b) demonstrated that the presence of phosphate still reduced BE substrate affinity, although to a lesser degree than with intact polyglucan substrates. All BEs showed substantially reduced substrate affinity with glycogen (Fig. S2), perhaps because the enzymes are attempting to work on a “finished” glycogen particle with reduced substrate availability. Furthermore, any possible effects of glucan phosphorylation on BE-substrate interactions may be diminished since previous studies indicate glucan phosphate tends to accumulate at the centre of the glycogen particle and may therefore be less accessible (Irimia et al., 2015; Nitschke et al., 2013). The effects of α-glucan phosphate appeared to be restricted to substrate binding and affinity rather than catalysis, since determination of BE catalytic activity by measuring changes in CLD indicated that the presence of α-glucan phosphate caused no observable change in branched products (Fig. S4). However, our data, which is based on CLD analysis of BE activity, should be treated with some caution, as this method requires long incubation times (5 h) of relatively large amounts of purified (recombinant) BE and glucan substrates in order to detect the products of BE activity under different treatments. A routinely used method for determining BE catalytic activity is the ^14^C-based phosphorylase *a*-stimulation assay which indirectly measures BE *via* its stimulation of GP-mediated chain elongation with lower incubation times (up to 90 min) (Smith, 1988). We considered this assay method unsuitable for testing the effects of α-glucan phosphate on BE catalytic activity because any possible effects of α-glucan phosphate would be confined to the initial stages of the reaction, and then masked by subsequent branching of new (non-phosphorylated) glucan chains created by phosphorylase *a*.

Our data suggests a common structural domain of BE responsible for substrate binding may be susceptible to the presence of phosphate esters on the α-1,4-glucan chains of these polyglucans. Most BEs are members of the glycoside hydrolase family-13 (GH13) of enzymes which includes ISA and α-amylase (AMY, EC 3.2.1.1), all sharing three functional domains of secondary structure, with eukaryotes belonging to subfamily-8, and bacteria subfamily-9 of GH13 (Stam, Danchin, Rancurel, Coutinho, & Henrissat, 2006). The BE domain most likely susceptible to substrate binding inhibition by α-glucan phosphate is the amino (N)-terminal region containing the conserved CBM48 glucan binding domain which holds BE to its substrate during the cleavage and transfer of α-1,4-glucan chains, and is distinct from the central, highly conserved, catalytic region of BEs (Devillers, Piper, Ballicora, & Preiss, 2003; Lawson et al., 1994). Interestingly, structural analyses of BEs predict a negatively charged substrate binding/catalytic pocket (Abad et al., 2002; Noguchi et al., 2011) which may in part account for the reduced interactions observed with phosphorylated glucan substrates in this study. The BEs used in this study show wide variation in their structural conservation (Table S2). It is interesting to note that the two BEs most susceptible to α-glucan-bound phosphate, *Sc*GLC3 and *Hs*GBE1 are most closely related in terms of structure (56 % homology).

Our *in vitro* observations suggests that phosphorylation of α-glucans during polymer biosynthesis impacts the subsequent actions of BEs, key enzymes which determine the structural and physicochemical properties of both starch and glycogen (Fig. 5a). As such, our data may provide the simplest interpretation of the well-documented phenomenon of the negative relationship between degree of branching and α-glucan phosphate levels in both plant starches and certain pathological states of eukaryotic glycogen metabolism. The biological significance of our findings is, at this stage, open to speculation, but it is possible to posit a role for α-glucan phosphate deposition during the biosynthesis of these two polymers in relation to indirect control of α-1,6-branch point deposition (see Fig. 5b-c). Our experiments were performed using potato amylopectin, which, together with other tuberous and rhizomatous starches represent the extremes of α-polyglucan phosphorylation, and therefore a useful baseline from which to conduct the glucan phosphate titration experiments described here. These high-phosphate starches are characterized by having relatively long glucan CLs in their amylopectin clusters (see Fig. 1c), perhaps because of higher expression levels of SBEI which *in vitro* branch longer glucan chains at low frequency relative to the SBEII class in these plants (Ahn et al., 2010; Guo et al., 2022; Qin, Zhou, & Zhang, 2013). Furthermore, many of the BEs we employed in this study likely encounter low, or in some cases, *e.g.* yeast and bacteria, perhaps no glucan phosphate, with many plant starches having relatively low glucan phosphate levels compared with tuberous starches (Blennow, Engelsen, et al., 2000; Fig. 1a). Therefore, the question of the biological significance of our findings is particularly salient. A tentative model is proposed which attempts to reconcile our observations with previously published data and suggest a role for α-glucan phosphate esters in determining aspects of both starch and glycogen structure (Fig. 5 b-c). The precise distribution and density of phosphate esters in starch and glycogen is not known, although it likely differs between the two polymers because of structural differences brought about by differing modes of biosynthesis. Both starch and glycogen are formed as a result of the balance between elongation activity by, respectively SS and GS, and branching activity by, respectively, SBE and GBE. In plants, SS and SBE activities are a result of multiple isoforms of each enzyme class, post-translational regulation, and formation of protein complexes between SSs and SBEs (Hennen-Bierwagen et al., 2008; Pfister et al., 2014; Tetlow et al., 2004; Tetlow et al., 2008). Furthermore, glucan branching frequency is further regulated by ISA1 and ISA2 to form a crystalline-competent, structurally complex polymer with densely clustered branch points with long, sparsely branched inter-connecting chains containing higher levels of α-glucan phosphate esters (Bertoft, Blennow, & Hamaker, 2024; Goren et al., 2018; Wikman et al., 2011), likely making the distribution of phosphate esters within the starch granule uneven. By contrast, the open, water-soluble structure of the glycogen particle requires relatively uniform branching frequency throughout the polymer structure *via* the actions of GS and GBE. The catalytic activity of GS is highly regulated (Roach et al., 2012), but it is not known if GBE is regulated at the post-translational level, nor whether it interacts with GS to regulate the ratio of elongation and branching during glycogen biosynthesis. Structural studies suggest that there is a higher density of α-glucan phosphate esters at the inner core of the glycogen particle (Irimia et al., 2015; Nitschke et al., 2013). Specifically, the model in Fig. 5b illustrates how localized phosphorylation of long, cluster-connecting glucan chains found in the amorphous regions of B-allomorph amylopectins may regulate inter-cluster spacing by inhibiting branching by SBEs and the formation of new clusters, creating the more open, hydrated, higher order structures which are not apparent in A-allomorph (storage) starches. Fig. 5c shows how glucan phosphate and branching of the glycogen particle may serve complementary roles in increasing and maintaining localized hydration (water-solubility) of glucan chains. Thus, the presence of phosphate may direct GBEs to regions of the polymer lacking branches and phosphate which are prone to insolubility.

## Supplementary Material

Supplemental information

## Data Availability

Data will be made available on request.

## Appendix A. Supplementary data

Supplementary data to this article can be found online at https://doi.org/10.1016/j.carbpol.2025.123561.
