# Supplementary material for: Covalently linked phosphate monoesters on alpha-polyglucans reduce substrate affinity of branching enzymes
Source: Carbohydr Polym. Author manuscript; Available in PMC 2026 Mar 24. (PMC13011383; doi:10.1016/j.carbpol.2025.123561)

**Supplementary Figures**


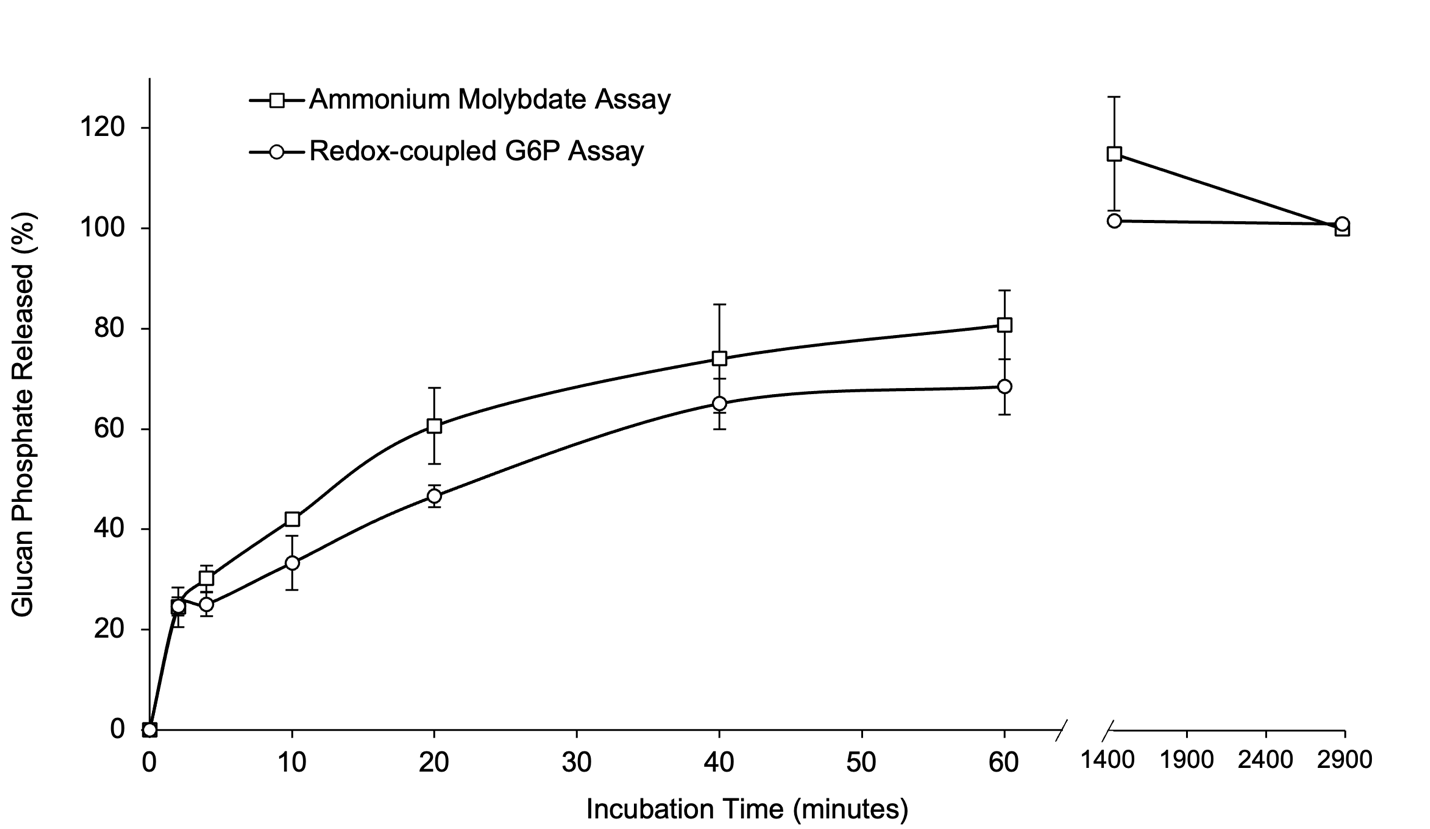


**Fig. S1.** Glucan phosphate cleaved (as a percentage of total glucan phosphate) from 20 mg potato amylopectin with 5 U of FastAP at 37°C over a 48-hour incubation period (n=3 ± S.D.). Hydrolytically cleaved inorganic phosphate was quantified with an ammonium molybdate assay (□) and remaining α-glucan-bound phosphate was quantified with an enzyme-coupled Glc6P assay (○). No significant difference was observed (Student’s *t*-test) between the amount of freed phosphate detected from each method.


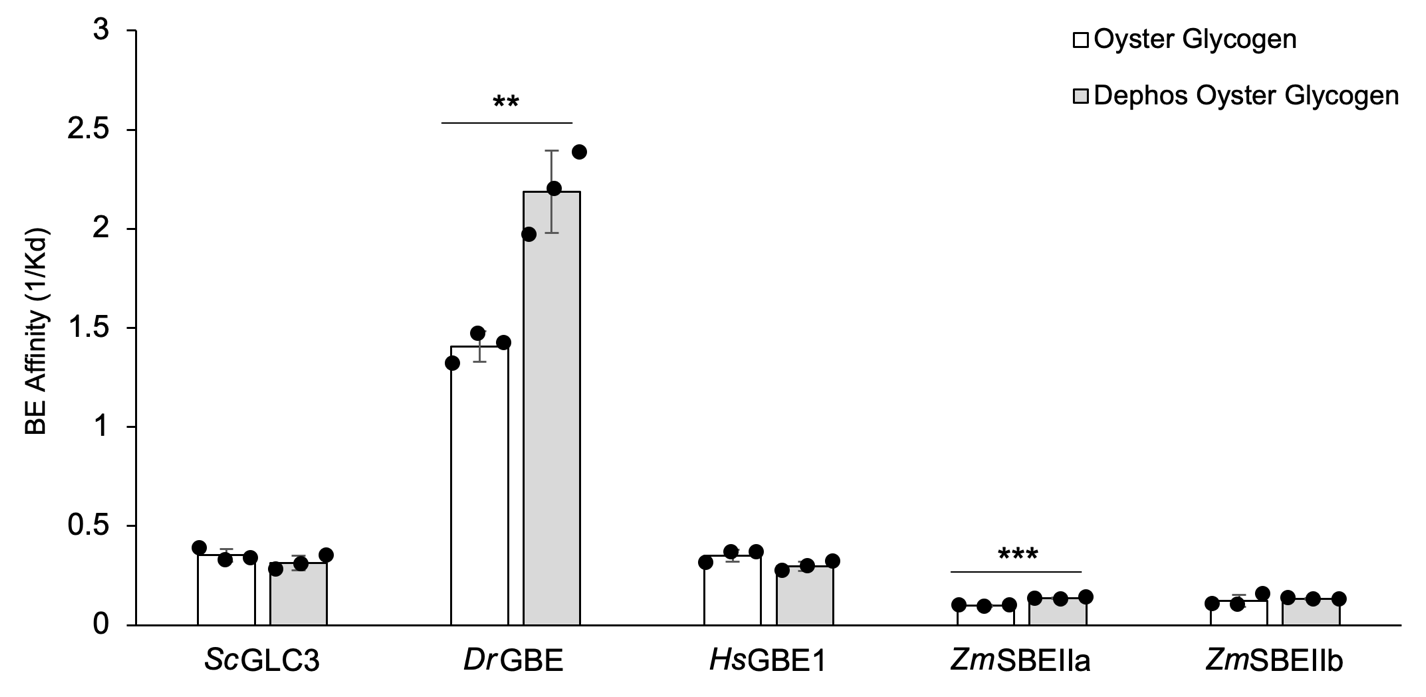


**Fig. S2.** Relative substrate affinity (1/*K*_d_) of different BEs for phosphorylated and dephosphorylated oyster glycogen. Untreated (phosphorylated) oyster glycogen contained ~1 mmol P/mg glucan. Data presented is the mean + S.D. of 3 replicates. **P<0.01, ***P<0.001.


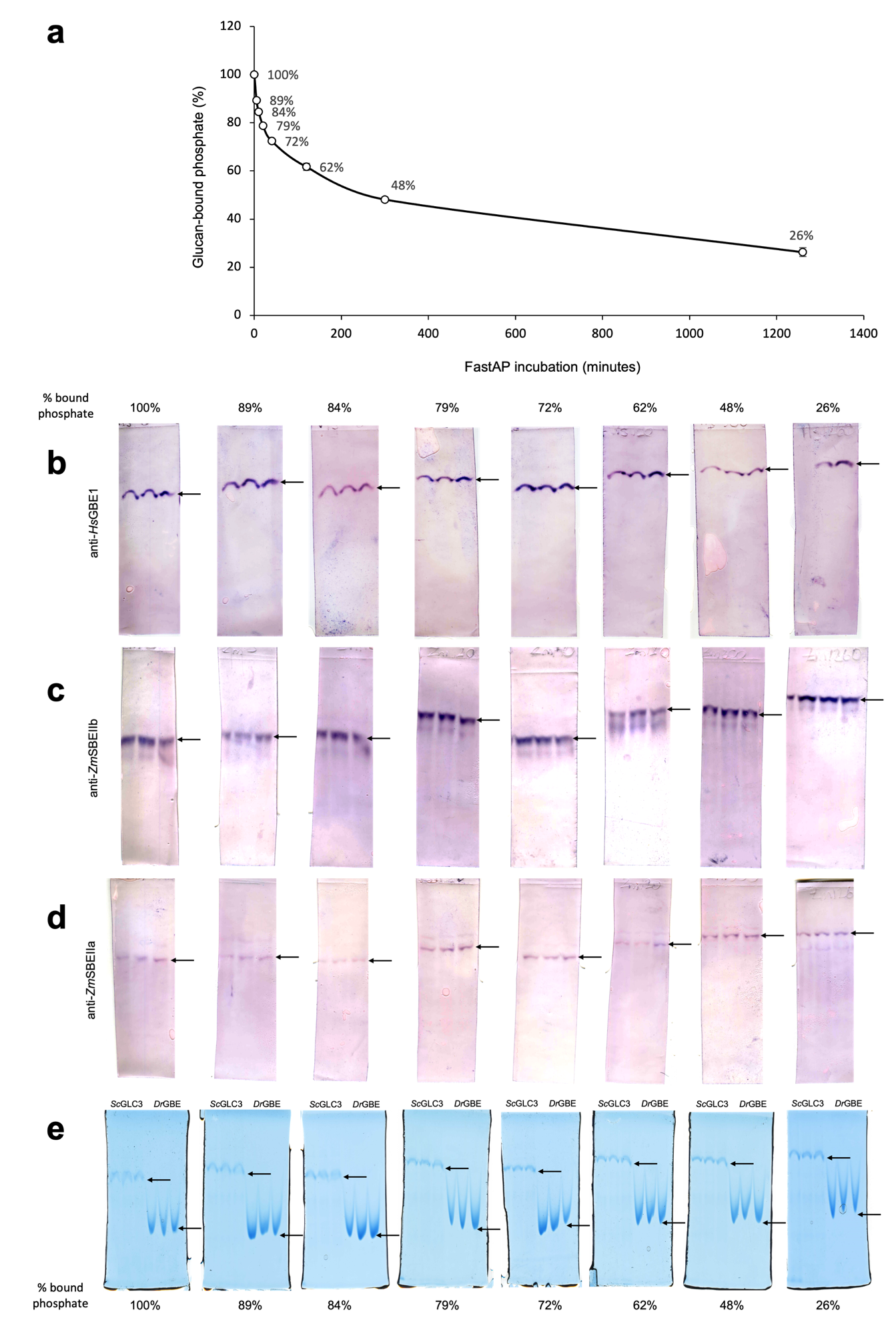


**Fig. S3.** Electrophoretic mobility of BEs in gels containing potato amylopectin with various degrees of glucan phosphorylation (26-100% glucan-bound phosphate) which coincides with titration of BE affinity presented in Fig. 4. **(a)** Kinetics of phosphate release from gelatinized potato amylopectin by FastAP over time, showing time points used and corresponding % glucan-bound phosphate remaining. 20 mg of gelatinized potato amylopectin was incubated with 10 U FastAP at 37°C for the designated times and stopped by boiling. Data presented is the mean + S.D. of 3 replicate experiments. (b-e) Potato amylopectin with varying levels of glucan-bound phosphate shown in (a) was used for BE affinity gel electrophoresis experiments shown in (b-e). Mobility of BEs (indicated by arrows) in gels containing variable % glucan-bound phosphate was visualized by western blotting of cell lysates (b-d) or Coomassie staining of purified recombinant enzymes (e) run through non-denaturing polyacrylamide gels. Nitrocellulose membranes probed with peptide specific antibodies for (b) *Hs*GBE1, (c) *Zm*SBEIIb, and (d) *Zm*SBEIIa, as well Coomassie stained gels of (e) *Sc*GLC3 and *Dr*GBE, display shifts in mobility due to changes in % glucan-bound phosphate.


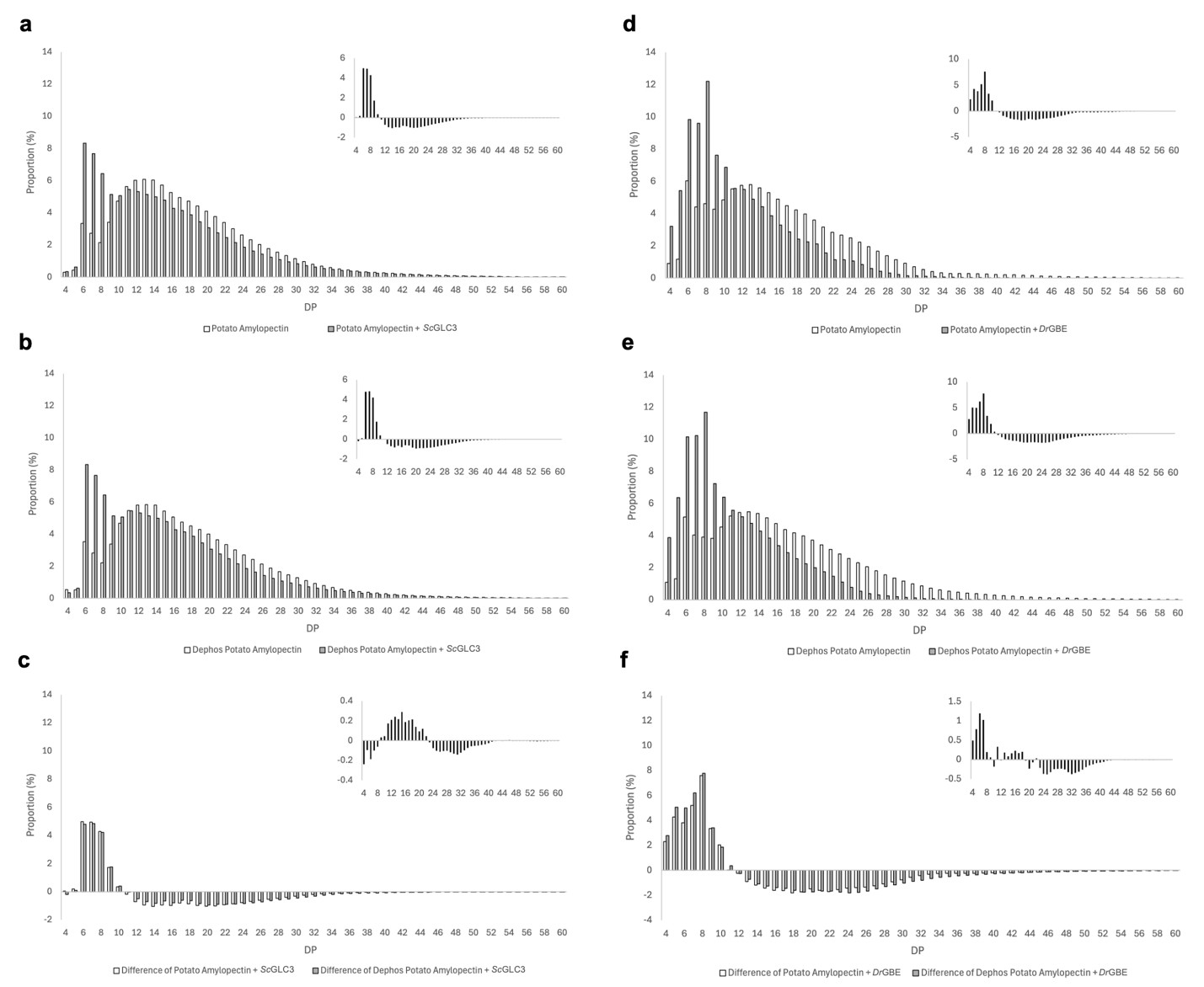


**Fig. S4.** Analysis of catalytic activities of *Sc*GLC3 and *Dr*GBE with phosphorylated and dephosphorylated potato amylopectin. Following incubation of BEs with gelatinized amylopectin, glucan products were debranched and the CLD profile analyzed by HPAEC-PAD. Data is expressed as the proportion (%) difference in moles of glucan chains produced following incubation with recombinant BE compared with the potato amylopectin substrate. Light bars are unmodified potato amylopectin, and dark bars are potato amylopectin treated with recombinant *Sc*GLC3 (a-c) or *Dr*GBE (d-f). Profiles a) and d) are phosphorylated potato amylopectin, profiles b) and e) are 100% dephosphorylated potato amylopectin. Insets for profiles a-e are difference plots showing proportional difference (%) between potato amylopectin and BE-treated potato amylopectin (BE-treated potato amylopectin minus potato amylopectin). Profiles c) and f) compare the CLD profile of BE-treated amylopectin *versus* BE-treated dephosphorylated amylopectin, with the insets showing the non-significant difference (<1%) between these profiles (BE-treated dephosphorylated amylopectin minus BE-treated potato amylopectin).


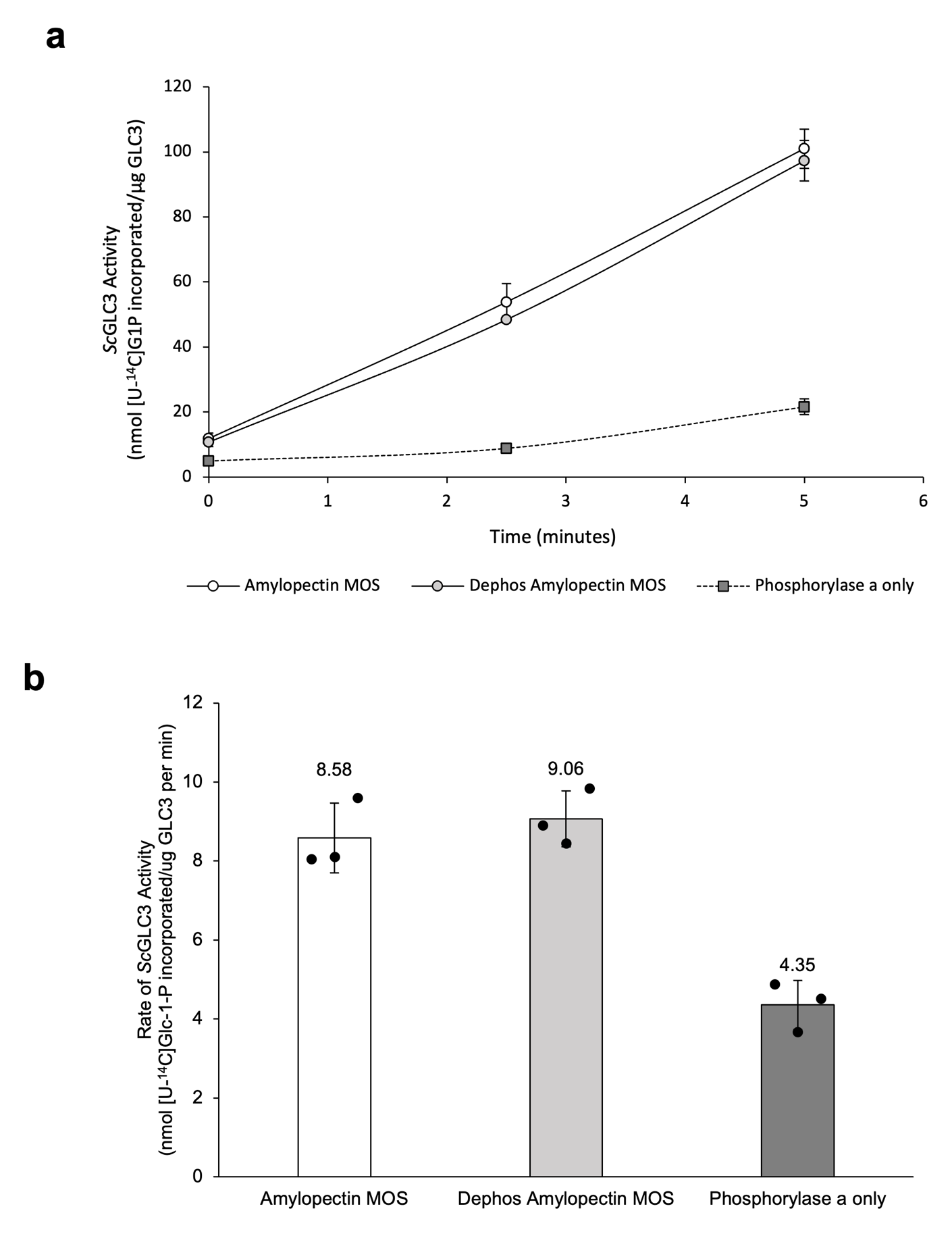


**Fig. S5.** Incorporation of [U-^14^C]-Glc1P by phosphorylase *a* stimulation assay with debranched potato amylopectin (Amylopectin MOS) and debranched dephosphorylated potato amylopectin (Dephos Amylopectin MOS) branched by *Sc*GLC3 and elongated by rabbit muscle phosphorylase *a*. (a) nmol of [U-^14^C]-Glc1P incorporated into amylopectin MOS (white circles), dephos amylopectin MOS (light grey circles), and phosphorylase *a* only (dark grey circles) over 5 mins (n=3 ± S.D.), (b) rate of *Sc*GLC3 activity for amylopectin MOS and dephos amylopectin MOS (n=3 ± S.D.), the difference of which was found to be non-significant by Student’s *t*-test.

**Supplementary Tables**

**Table S1.** Average CL of HPAEC-PAD separated potato amylopectin (regular and dephosphorylated) as well as *Sc*GLC3 and *Dr*GBE treated potato amylopectin from Fig. S5. CL was determined according to the methods of Annor et al. 2014 and Bertoft et al. 2008, in which average chain length = sum of weight % of glucan sample/sum of glucan moles in sample. Changes in average CL between phosphorylated and dephosphorylated potato amylopectin was not found to be significant.

| **Polyglucan** | **Average CL** |
| --- | --- |
| Potato Amylopectin | 17.6 |
| Dephosphorylated Potato Amylopectin | 17.8 |
| Potato Amylopectin + *Sc*GLC3 | 15.3 |
| Dephosphorylated Potato Amylopectin + *Sc*GLC3 | 15.3 |
| Potato Amylopectin + *Dr*GBE | 11.4 |
| Dephosphorylated Potato Amylopectin + *Dr*GBE | 11.1 |

**Table. S2.** Sequence identity (%) matrix of BE orthologs from various organisms. Sequence identity was determined using EMBOSS Needle Pairwise Sequence Alignment. Sequences aligned in descending order are: Yeast (*Saccharomyces cerevisiae*) GBE (*Sc*GLC3), human (*Homo sapiens*) GBE (HsGBE1), *Escherichia coli* GBE (*Ec*GlgB), *Deinococcus radiodurans* GBE (*Dr*GBE), maize (*Zea mays*) SBEI (*Zm*SBEI), maize SBEIIa (*Zm*SBEIIa), maize SBEIIb (ZmSBEIIb), potato (*Solanum tubersoum*) SBEI (*St*SBEI), potato SBEII (*St*SBEII), Arabidopsis (*Arabidopsis thaliana*) SBE2.1 (*At*BE2.1), and Arabidopsis SBE2.2 (*At*BE2.2). *Sc*GLC3 shares highest sequence identity with *Hs*GBE1 (56%, green shaded box), followed by the plant SBEs (41.2-45.2% for maize and Arabidopsis SBEs, yellow shaded box, and 39-39.4% for potato SBE, orange shaded box), and bacterial GBEs (22-22.3%, red shaded box).


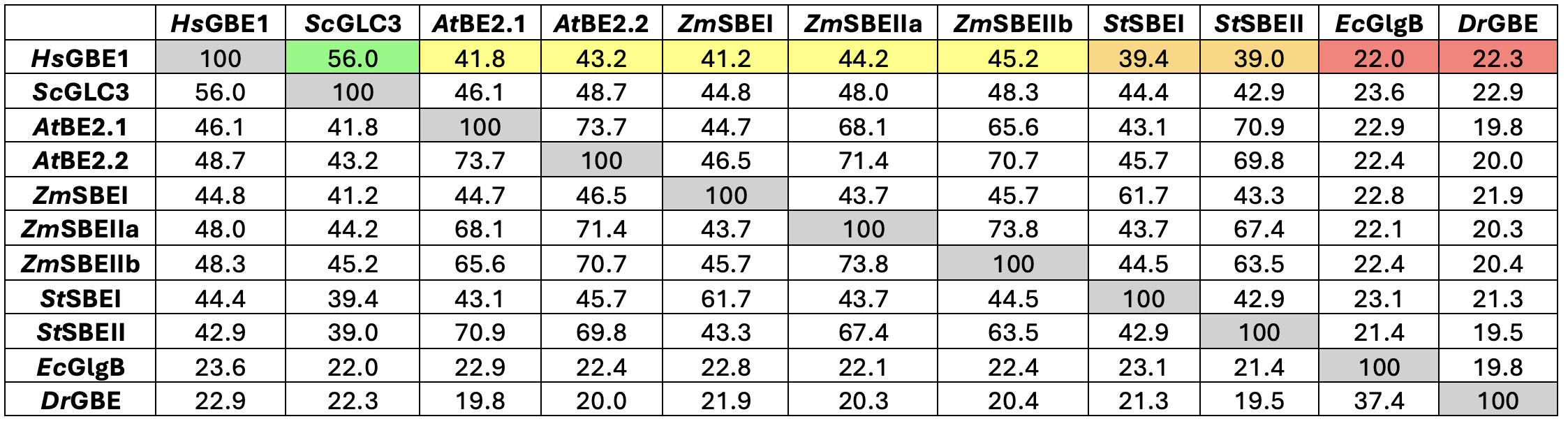

Supplement: Supplemental information [file NIHMS2151622-supplement-Supplemental_information.docx]
